# A Unifying Theory of Branching Morphogenesis

**DOI:** 10.1016/j.cell.2017.08.026

**Published:** 2017-09-21

**Authors:** Edouard Hannezo, Colinda L.G.J. Scheele, Mohammad Moad, Nicholas Drogo, Rakesh Heer, Rosemary V. Sampogna, Jacco van Rheenen, Benjamin D. Simons

**Affiliations:** 1 Cavendish Laboratory, Department of Physics, University of Cambridge, Cambridge CB3 0HE, UK; 2 The Wellcome Trust/Cancer Research UK Gurdon Institute, University of Cambridge, Cambridge CB2 1QN, UK; 3 The Wellcome Trust/Medical Research Council Stem Cell Institute, University of Cambridge, Cambridge CB2 1QN, UK; 4 Cancer Genomics Netherlands, Hubrecht Institute-KNAW and University Medical Centre Utrecht, Utrecht 3584CT, the Netherlands; 5 Northern Institute for Cancer Research, Newcastle University, Newcastle upon Tyne NE2 4AD, UK; 6 Department of Biomedical Engineering, University of Rochester, Rochester, NY 14627, USA; 7 Division of Nephrology, Department of Medicine, Columbia University College of Physicians and Surgeons, New York, NY 10032, USA

**Keywords:** branching morphogenesis, mammary gland, kidney, prostate, mathematical modeling, branching and annihilating random walks, self-organization

## Abstract

The morphogenesis of branched organs remains a subject of abiding interest. Although much is known about the underlying signaling pathways, it remains unclear how macroscopic features of branched organs, including their size, network topology, and spatial patterning, are encoded. Here, we show that, in mouse mammary gland, kidney, and human prostate, these features can be explained quantitatively within a single unifying framework of branching and annihilating random walks. Based on quantitative analyses of large-scale organ reconstructions and proliferation kinetics measurements, we propose that morphogenesis follows from the proliferative activity of equipotent tips that stochastically branch and randomly explore their environment but compete neutrally for space, becoming proliferatively inactive when in proximity with neighboring ducts. These results show that complex branched epithelial structures develop as a self-organized process, reliant upon a strikingly simple but generic rule, without recourse to a rigid and deterministic sequence of genetically programmed events.

## Highlights

• Branching morphogenesis follows conserved statistical rules in multiple organs
• Ductal tips grow and branch as default state and stop dividing in high-density regions
• Model reproduces quantitatively organ properties in a parameter-free manner
• Shows that complex organ formation proceeds in a stochastic, self-organized manner

## Graphical Abstract

**Figure:**
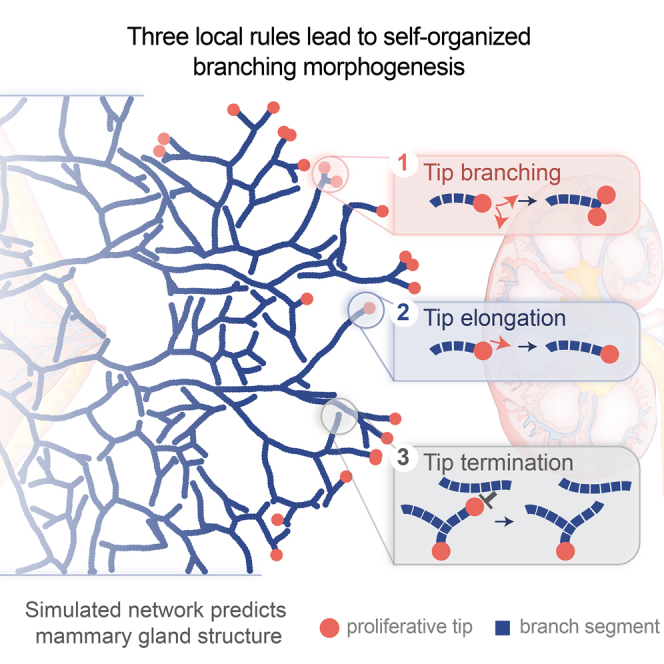

## Teaser

Complex branched epithelial structures in mammalian tissues develop as a self-organized process, reliant upon a simple set of local rules.

## Introduction

Branching morphogenesis has fascinated biologists and mathematicians for centuries, both because of its complexity and ubiquity (Hogan, 1999, Lu and Werb, 2008, Metzger et al., 2008). In higher organisms, many organs are organized into ductal tree-like structures comprising tens of thousands of branches, which typically function to maximize the surface of exchange between the epithelium and its lumen. Examples include lung, kidney, prostate, liver, pancreas, the circulatory system and the mammary gland epithelium. Alongside metazoa, tree crowns and root systems as well as coral reefs often display a similar branched organization (Harrison, 2010) raising the question of whether common mechanisms could underlie their formation. Extensive investigations have identified features shared by all branched organs, which are formed by repeated cycles of branching (either through side-branching or tip-splitting), together with phases of ductal elongation (Iber and Menshykau, 2013).

Attempts to resolve the regulatory basis of branching morphogenesis have been targeted at different length scales, offering contrasting perspectives. First, at the molecular scale, key regulatory signaling pathways, for instance controlling proliferation and cell fate, have been resolved in multiple organs (Iber and Menshykau, 2013). Second, at the cellular and mesoscopic scale, measurements of gene expression patterns and branching shape have implicated Turing-like mechanisms in the regulation of the first rounds of repetitive branching in the lung and kidney (Iber and Menshykau, 2013, Guo et al., 2014). Alternative, potentially overlapping, explanations based on mechanical (Gjorevski and Nelson, 2011) or viscous (Lubkin and Murray, 1995) models have been proposed, and processes such as oriented cell divisions (Yu et al., 2009), collective cell migration (Huebner et al., 2016, Riccio et al., 2016), and cytoskeleton-driven cell shape changes (Elliott et al., 2015, Kim et al., 2015) have been shown to play a role. Yet, even a perfect understanding of how single branching events occur would not explain how thousands of tips and branches become coordinated at the organ scale, to specify a complex ductal network.

Therefore, here, we adopt an alternative “non-reductionist” approach and test whether the statistical properties of branched networks can be predicted without extensively addressing the detailed underlying molecular and cellular regulatory processes. Historically, the development of such statistical approaches has been limited by the lack of high resolution biological data on the complete organ structure. However, this problem is becoming alleviated by advances in imaging techniques (Metzger et al., 2008, Sampogna et al., 2015, Short et al., 2014), which provide an ideal platform to question how a complex 3D organ structure is encoded. Does it form from the unfolding of an intrinsic deterministic program or is it shaped by extrinsic influences and stochastic processes?

In the following, we use detailed whole-organ imaging and 3D reconstructions of the mouse mammary gland epithelium, mouse kidney, and human prostate to address the spatiotemporal dynamics of branching morphogenesis. We show that the detailed statistical properties of these organs share key underlying features, which can be explained quantitatively through a remarkably simple and conserved design principle, based on the theory of branching and annihilating random walks (BARWs). In this model, growing ductal tips follow the same, time-invariant, statistical rules based on stochastic ductal branching and random exploration of space. However, when an active tip comes into proximity with a neighboring duct, it becomes irreversibly inactive (differentiating and exiting cell cycle), leading to the termination of the duct. We show that, together, these simple local rules are enough to allow the epithelium to grow in a self-organized manner, into a complex ductal network with conserved statistical properties that are quantitatively predicted by the model. Notably, these isotropic rules predict the emergence of directional bias in the expansion of the ductal network, in the absence of any external guiding signaling gradients. Finally, to further challenge the model, we predict, and discover experimentally, novel signatures of the inferred dynamics, which are consistent with an out-of-equilibrium “phase transition.” Moreover, by adjusting experimentally the microenvironment of the branching tips through local or systemic perturbations, we further test the predictive capacity of the model and gain insight into the molecular regulatory basis of the inferred collective cell dynamics.

## Results

### Defining the Ductal Network Structure of the Mouse Mammary Gland Epithelium

**Figure S1:**
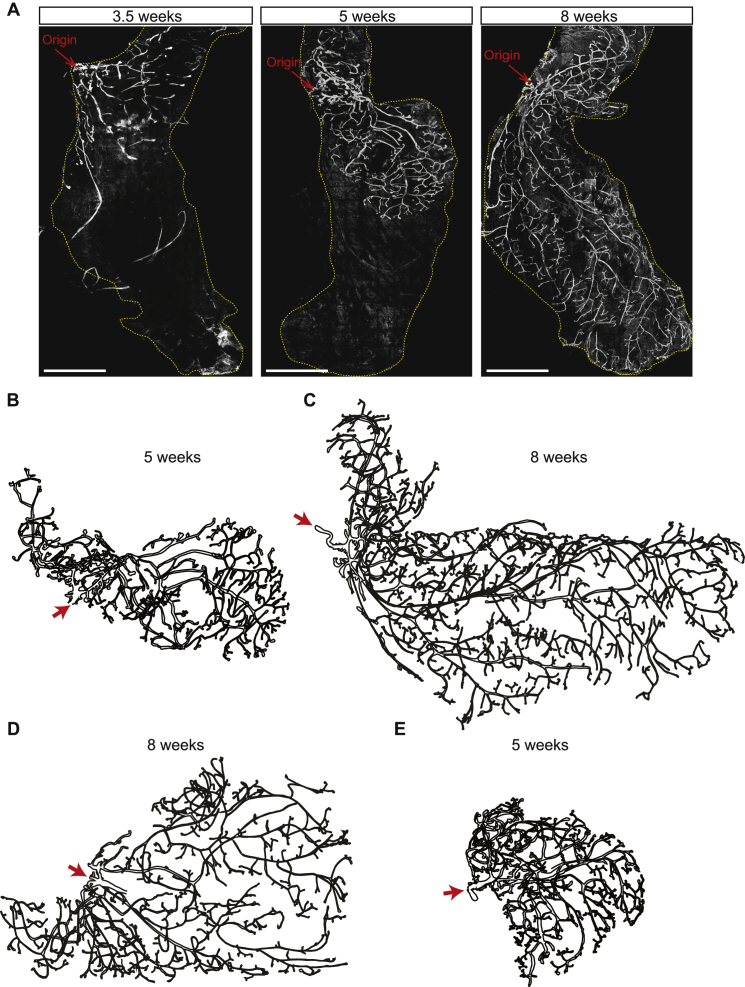
Whole-Gland Reconstructions Reveal the Dynamics of Fat Pad Invasion and Branching Morphogenesis, Related to Figure 1 (A) Whole-gland imaging (K14 staining in white) of fourth mammary glands of 3.5w, 5w and 8w old mice. Pubertal morphogenesis starts from a rudimentary tree around 3.5w and is complete around 8w. (B) Post-reconstruction outlines of the 5w old mammary glands seen in (A). (C) Post-reconstruction outlines of the 8w old mammary gland seen in (A). (D) Post-reconstruction outlines of another 8w old fourth mammary gland reveals the heterogeneity and stochasticity underlying mammary gland formation and structure. (E) Post-reconstruction outlines of a 5w old fifth mammary gland shows a similar structure and organization compared to fourth mammary glands. Gland origin indicated in each panel by red arrow. Scale bars: 5 mm.

To develop a model of tip-driven ductal morphogenesis, we began by considering the mammary gland epithelium. At birth, the mouse mammary gland is specified as a small rudimentary tree-like structure (Figure S1A). During puberty, precursors localized at ductal tips (termed terminal end-buds) drive the expansion of a complex network through multiple rounds of tip bifurcation and ductal elongation (Sternlicht, 2006) (Figure S1A). These networks are characterized by the structural heterogeneity of their subtrees (that we defined formally as the parts of the ductal tree sharing a common branch ancestor at branch level *l*_e_ = 6—that reflects the approximate extent of the rudimentary structure prior to pubertal morphogenesis). Indeed, some ductal subtrees become extremely large, containing as many as 30 generations of consecutive branching events, while neighboring subtrees may terminate precipitously (Scheele et al., 2017).

Recently, we showed through quantitative genetic lineage tracing methods that the complexity of the mammary epithelium does not derive from intrinsic heterogeneity of tip precursor populations, but from the stochastic fate decisions of equipotent tips, which either branch (bifurcate) or terminate (through cell-cycle exit) with near equal-probability (Scheele et al., 2017), suggestive of a local control of tip fate. However, such a focus on spatially averaged models of branching morphogenesis (Zubkov et al., 2015) cannot resolve the spatiotemporal dynamics and mechanistic basis of the underlying regulatory program, nor its potential conservation in other organs.

**Figure 1:**
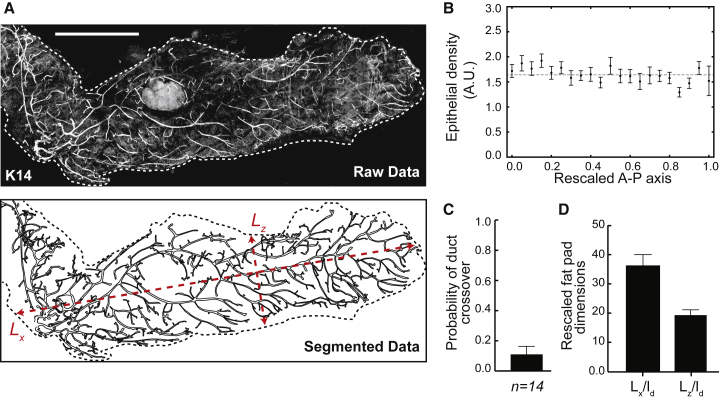
Geometry and Characteristics of Murine Mammary Glands Revealed by Quantitative Reconstructions (A) Quantitative reconstruction (top) and outline (bottom) of a fourth mammary gland based on Keratin14 (K14) staining (white), reproduced from (Scheele et al., 2017) along with measurements of the fat pad dimensions *L*_x_ and *L*_z_ (red). (B) Density profile of ducts along the rescaled antero-posterior axis. (C) Counting of ductal crossovers (normalized by total number of ductal branches) reveal a low crossing probability. (D) Experimentally measured ratio between the dimensions of the mammary fat pad (long axis *L*_x_ and short axis *L*_z_) and the average length of a branch *l*_d_, used for the simulations of the mammary gland. Error bars represent mean and SEM. Scale bar, 5 mm. See also Figure S1.

How can such a balance between tip termination and branching be regulated at the population level? One possibility is that tip branching and termination rates are dependent on the local epithelial density. Indeed, an increase in the termination rate and/or a decrease in the branching rate with local density can ensure that the system reaches a robust steady state, characterized by a balance between tip branching and termination (Method Details). Interestingly, such behavior generically produces glands of uniform spatial density, a feature that we verified experimentally by reconstructions of whole adult mammary glands (n = 14 glands, Figures 1A and 1B). However, to understand whether it is branching or termination events that are actively regulated, we turned to quantitative measurements. In particular, parameterization of the distribution of mammary branch lengths (defined as the distance between consecutive branching points) revealed a strikingly exponential dependence, with an average branch length that remains approximately constant over time (Scheele et al., 2017). This observation suggests that the timing between consecutive branching events is random and statistically uncorrelated, pointing to a stochastic and time-invariant program of tip branching. This behavior stands in stark contrast with the early stages of lung morphogenesis, where branch lengths for a given branching level are tightly controlled (Iber and Menshykau, 2013).

We then examined the spatial organization of the ductal network. As the mammary fat pad constrains growth to a thin pancake-like geometry, ductal morphogenesis of the mammary epithelium takes place in a near 2D setting. Against this background, inspection of whole gland reconstructions revealed a strikingly low frequency of ductal crossovers (Figures 1A, 1C, 1D, and S1) with terminated tips often residing close to an existing duct or the fat pad boundary (Silberstein, 2001). This observation suggests that ductal elongation and branching may proceed as a “default state,” with tip termination occurring only when tips come into proximity with existing ducts. Such behavior is consistent with *in vitro* measurements (Nelson et al., 2006), which show that ductal branching only occurs when remote from the other ducts, with tips close to neighbors remaining inactive.

### Mammary Morphogenesis Proceeds as a Branching and Annihilating Random Walk

**Figure 2:**
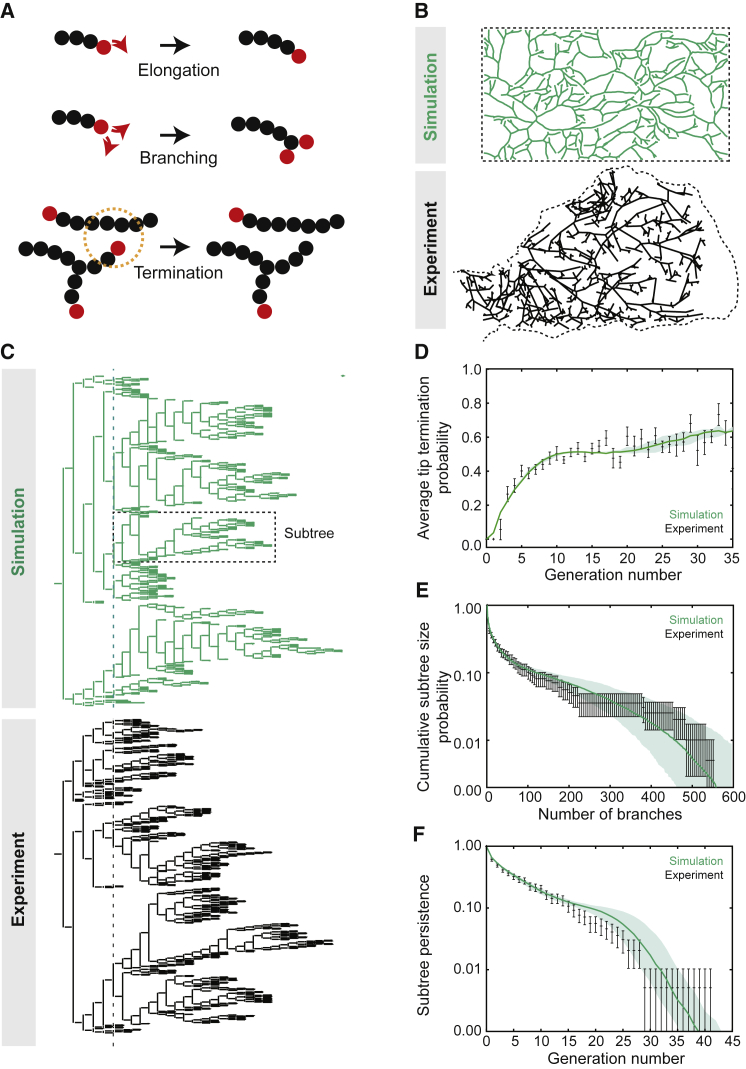
A Model Based on Branching and Annihilating Random Walks Predicts Quantitatively Mammary Branching Morphogenesis (A) Schematic of the model. Active ductal tips choose between ductal elongation, stochastic branching through tip bifurcation, or termination when in proximity to a neighboring duct. (B) Comparison between the experimental and theoretical structure of mammary glands. (C) Comparison between the experimental and theoretical topology of the trees, displaying large heterogeneity, with different subtrees (defined as parts of the tree starting at level 6, delineated as dashed line, with a black box showing an example of a subtree) growing to widely different sizes. (D–F) The BARW model predicts quantitatively the evolution of the probability for tips to terminate (D), the cumulative distribution of subtree size (E), and the subtree persistence to a given branch generation number (F). Data from Scheele et al. (2017). Shaded area and error bars in (E and F) represent mean ± 1 SD confidence intervals. Error bars in (D) represent mean and SEM. Black represents experiments and green theoretical predictions from simulations. See also Figure S2 and Movie S1.

Interestingly, such a model of branching morphogenesis maps directly onto the theory of “branching and annihilating random walks,” a class of models studied extensively by physicists (Cardy and Täuber, 1996) showing that the dynamics of binary tip-splitting models converge over time onto a common statistical behavior belonging to the universality class of “directed percolation.” Here, we implemented a minimal model of branching morphogenesis, inspired by the theory of BARWs, where tip dynamics involves only three processes (depicted in Figure 2A): (1) ducts elongate from active tips in a random direction with a speed *v*—“a persistent random walk”—leaving behind a trail of static, non-proliferative ducts; (2) at any instant, ducts can branch through stochastic tip bifurcation with a constant probability *r*_b_; and (3) ducts terminate through tip inactivation when tips come within an annihilation radius *R*_a_ of an existing duct.

**Figure S2:**
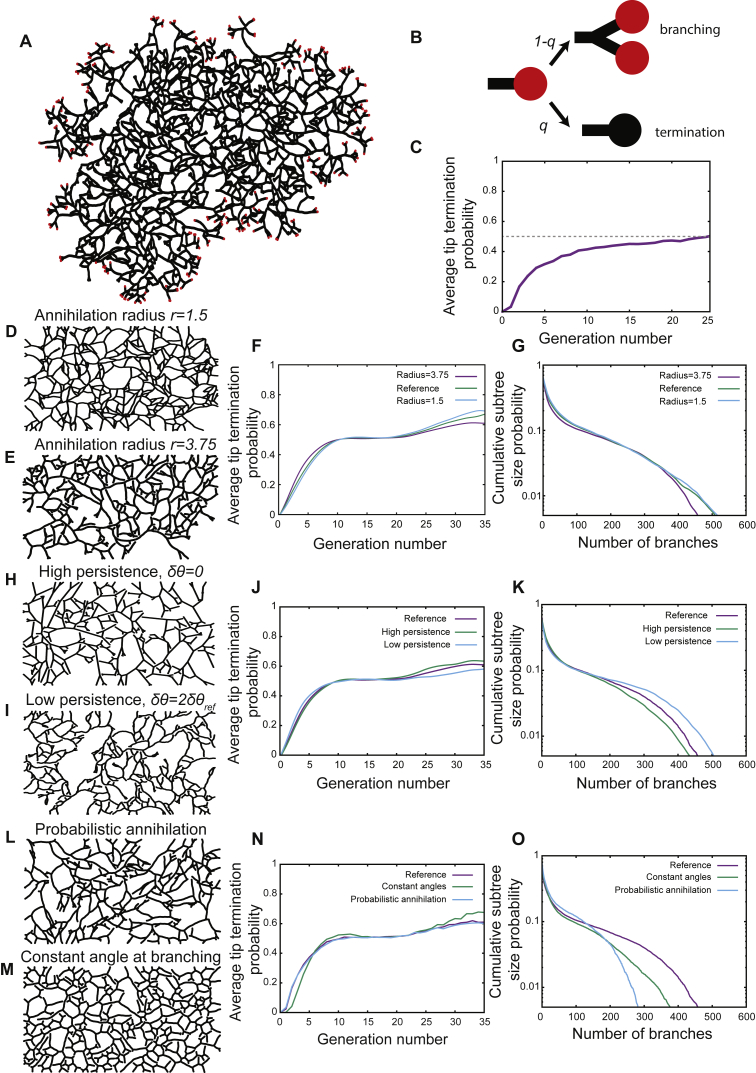
Branching and Annihilating Random Walks Robustly Reproduce the Experimental Phenomenology, Related to Figure 2 (A) Typical output of a numerical simulation of our model, using the same parameters used for the wild-type case of Figure 2, but in an unbound geometry. Active tips are color-coded in red, while inactive ducts are in black. (B) Definition of the tip termination probability *q*, and the converse probability to branch *1−q*. In the following we present termination probabilities averaged for all tips of a given generation number. (C) Average tip termination probability from an average of 500 simulations in an unbound domain shown in A, as a function of the generation number. This demonstrates a robust convergence toward a balance between tip termination and tip branching (green horizontal line). (D–G) Sensitivity of the results of the model as a function of the annihilation radius *R*_*a*_. We perform simulations with a smaller radius (*R*_*a*_ = 1.5, panel D) and a larger radius (*R*_*a*_ = 3.75, panel E), and we compare in each case the termination probability (F) and subtree size distribution (G) to show that the results are only very weakly dependent on *R*_*a*_. (H–K) Sensitivity of the results of the model as a function of the persistence angle *δ θ*. We perform simulations with an infinite persistence (*δ θ* = 0, panel H) and a persistence halved from wild-type (*δ θ* = 2*δ θ*_*re f*_, panel I), and we compare in each case the termination probability (J) and subtree size distribution (K) to show that the results are only weakly dependent on *δ θ*. (L–O) Numerical simulations for a probability annihilation (i.e., instead of deterministic as in the wild-type simulation, L), or for a constant angle upon branching (M). We compare in each case the termination probability (N) and subtree size distribution (O). We find that the results of the tip termination probability are largely unaffected in all conditions, showing a robust convergence toward 0.5 on the same timescales. Probabilistic annihilation however markedly changes the shape of the subtree size distribution, as discussed in the STAR Methods.

Significantly, numerical simulations of the model dynamics in the absence of physical boundary constraints shows that the system reaches robustly a non-equilibrium steady state in which the frequency of branching and termination events becomes naturally balanced (Figures S2A and S2B; Method Details). As tips are not observed to cross the boundary of the fat pad and frequently terminate in their proximity (Figure 1A), we further implemented simulations of branching morphogenesis in a rectangular box of length *L*_x_ and width *L*_z_ to mimic these geometric constraints. Thus, the only key parameter of the model is the ratio between the dimensions of the fat pad and the average branch length *l*_d_ (the latter fixed by the ratio *v*/*r*_b_) (Figure 1D). Indeed, this geometrical parameter was fitted to its measured value (Figure S2; Method Details), so all subsequent comparisons with experiment represent the result of model predictions that do not involve the adjustment of any free parameter.

While a visual inspection of a typical simulation output revealed good qualitative agreement between the experiment and the theoretical predictions of the spatial organization (Figure 2B) and topology (Figure 2C) of the mammary ductal network, can such a simple model dynamics also provide quantitative insights? To address this question, we first quantified how the predicted frequency of tip bifurcation versus termination events evolves with branch level (i.e., the number of generations since the origin). Interestingly, as well as recapitulating long-term balance in the frequency of tip bifurcation and termination, we found that the model faithfully reproduced the dynamics of convergence toward balance, from an initial stage of symmetric branching early in pubertal development, where the ductal density is low (Figure 2D, *R*^2^ = 0.73). Strikingly, the model also predicted with high precision the heterogeneity of subtrees in mammary glands (defined in Figure 2C), quantified both by the subtree size distribution (Figure 2E, *R*^2^ = 0.96) and the subtree persistence to a given level (Figure 2F, *R*^2^ = 0.99).

**Figure S3:**
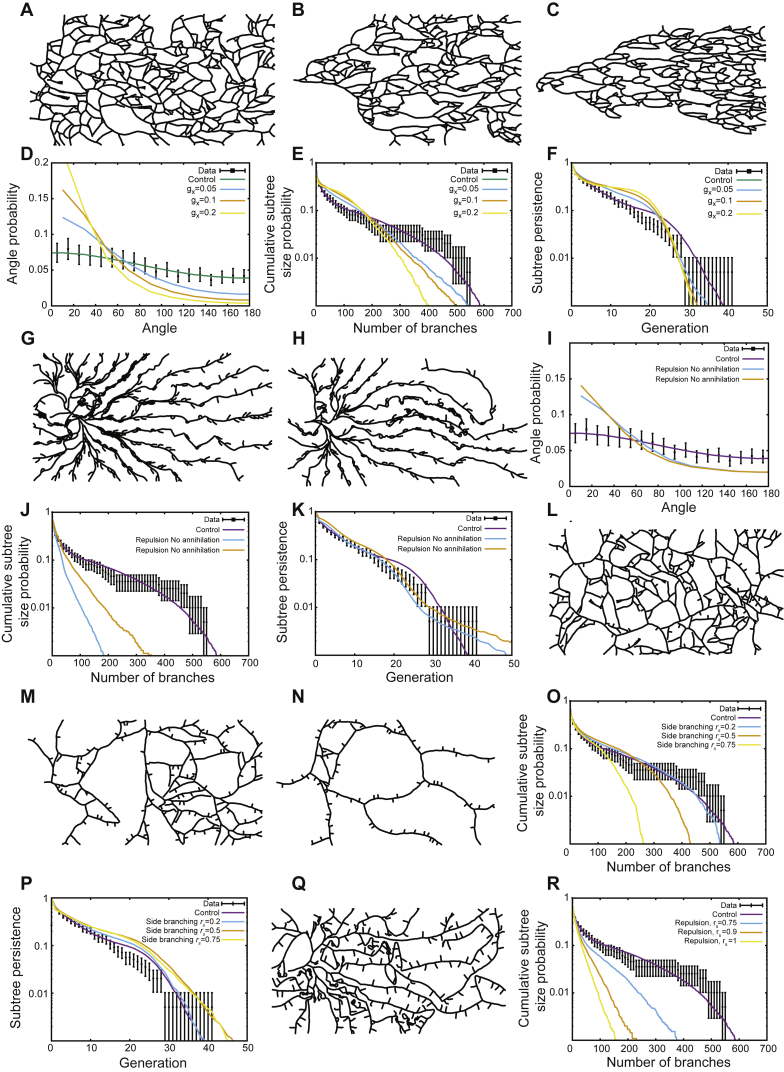
Alternative Models of Branching Morphogenesis Do Not Fit the Experimental Data, Related to Figure 2 (A–F) Typical output of numerical simulations of our model (A–C), and corresponding statistics (D–F), using the same parameters as used for the wild-type case of Figure 2, but with a guiding gradient that orients tips toward the distal side with strengths of *g*_*x*_*= 0.05* (A and blue curves), *g*_*x*_*= 0.1* (B and orange curves) and *g*_*x*_*= 0.2* (C and yellow curves). In each case, we compare the data (black) to the default model of Figure 2 (purple), and the predictions from the simulations with a gradient, for the branch angle probability (D), cumulative subtree size distribution (E) and subtree persistence (F). (G–K) Typical output of numerical simulations of our model (G and H), and corresponding statistics (I–K), using the same parameters used for the wild-type case of Figure 2, but with tip-duct repulsion (repulsion radius of *R*_*a*_ = *L*_*x*_/20 and a repulsion strength *f*_*r*_*= 0.6*) and medium (G and orange curve) and large (H and blue curve) side-branching (see STAR Methods for details). In each case, we compare the data (black) to the default model of Figure 2 (purple), and the predictions from these simulations for the cumulative subtree size distribution (J) and subtree persistence (K). (L–P) Typical output of numerical simulations of our model (L–N), and corresponding statistics (O and P), using the same parameters used for the wild-type case of Figure 2, but with various probabilities of side-branching: *r*_*s*_ = 0.2 (L and blue curves), *r*_*s*_ = 0.5 (M and orange curves) and *r*_*s*_ = 0.75 (N and yellow curves), see STAR Methods for details. In each case, we compare the data (black) to the default model of Figure 2 (purple), and the predictions from these simulations for the cumulative subtree size distribution (O) and subtree persistence (P). (Q and R) Typical output of numerical simulations of our model (Q), and corresponding statistics (R), using the same parameters used for the wild-type case of Figure 2, but with self avoidance between tips and ducts (repulsion radius of *R*_a_ = *L*_*x*_/60 and a repulsion strength *f*_r_ = 0.6), and various probabilities of side-branching (*r*_*s*_ = 0.75, blue curve, *r*_*s*_ = 0.9, [Q] and orange curves, *r*_*s*_ = 1, yellow curves; see STAR Methods for details). In each case, we compare the data (black) to the default model of Figure 2 (purple), and the predictions from these simulations for the cumulative subtree size distribution (R).

**Figure S4:**
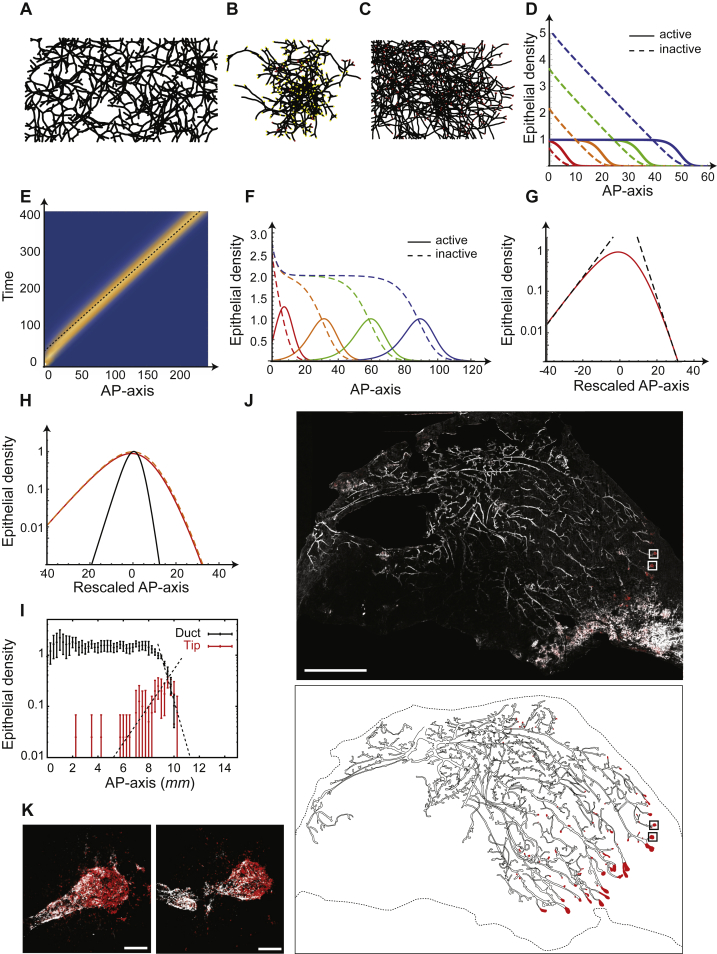
Numerical Simulations of Branching and Annihilating Random Walks Are Captured by a Fisher-KPP Mean-Field Theory, Related to Figure 3 (A) Typical simulation output for branching and annihilating random walks with a regulation on the branching probability, and constant and uniform annihilation probability. Contrary to the default case, although the model succeeds in reproducing the constant ductal density along the AP-axis, numerous cross-overs can be seen in the simulation outputs. (B) Typical simulation output for intrinsically balanced tip termination and branching, irrespective of local cues. This displays cross-overs and lack of space filling properties. (C) Typical simulation output for classical branching and annihilating random walks where tip annihilation only occurs in contact to another active tip. Active tips are uniformly distributed along the gland, instead of sitting at the front of the invasion, and the ductal density displays a gradient, together with numerous cross-overs between ducts. (D) The key aspects of (C) are well-captured by a mean field Fisher-KPP theory, for which we show a typical numerical integration output (rescaled branching rate $\bar{r}$ *= 1*), for different time points (red, orange, green and blue represent successive times). The full lines represent the density of active tips, while the dashed lines represent the density of inactive particles, i.e., ducts. (E) Kymograph of the numerical integration of Figure 3B, showing the propagation of a solitary pulse of active tips, traveling at constant density (rescaled branching rate $\bar{r}$ *= 0.1*). The dashed line represents the analytical prediction for a speed of *V = 2*$\bar{r}$^*1/2*^, which fits very well the numerical simulation. (F) Stationary shape of the KPP pulse in the same numerical simulation as E/, with the x axis rescaled around 0 (red). The dashed black lines represent of the analytical prediction for the exponential decay length of the back and front of the front, fitting very well with the numerical simulations. (G) Numerical integration of the Fisher-KPP equations, with the same parameters as Figure 3B, but making the approximation discussed in the STAR Methods that i≪a and $\bar{ra\left(a+i\right)\ll }a$. We observe very similar results, validating the approximation scheme, for different time points (red, orange, green and blue represent successive times). The full lines represent the density of active tips, while the dashed lines represent the density of inactive particles, i.e., ducts. (H) Stationary shape of the KPP pulse, comparing three conditions: numerical integration of the full KPP equations as in (D) (full red line, $\bar{r}$ *= 0.1*), approximation discussed in (F) (dashed orange line, $\bar{r}$ *= 0.1*), and numerical integration of the full KPP equations for larger $\bar{r}$ *= 1*, where the approximation becomes invalid (thin black line). For $\bar{r}$ *= 0.1*, one notices that the approximation matches very well the integration of the full equation. Increasing $\bar{r}$ *= 1* produces sharper pulses as expected, but still leads to the same phenomenology discussed in the $\bar{r}$*→ 0* limit of a pulse which decays slower in the back that in the front. (I) Experimental density of ducts (black) and proliferative tips (same as Figure 3D), overlaid with the fitted analytical prediction of an exponential decay at the front for both tips and ducts (dashed line). Importantly, the back of the pulse was well-fitted by the theoretical prediction of a slope √2 − 1 (less steep, dashed line). (J) Reconstruction of a 5w third mammary gland following an Edu pulse, showing a similar result as Figure 3C, with a pulse of tips at the invasion front and large density fluctuations. (K) Zoom on representative terminal end bud structures, i.e., active tips containing highly proliferative cells, from the boxes shown on I. Error bars represent mean and s.e.m. Scale bars: 5 mm (I) and 100μ m (J). K14 in white, EdU in red.

Importantly, the spatial model accounted more accurately for the abundance of very large subtrees, which appear due to spatial “priming” in low density regions, than the previously published “zero-dimensional” model (Scheele et al., 2017) in which tip branching and termination events are defined intrinsically and probabilistically. More generally, to explore the specificity of the model, we also considered the quantitative predictions made by eight further classes of models, corresponding to various alternative proposals from the literature. In each case, their applicability to the experimental data was found to be limited (see Figures S3 and S4A–S4D; Method Details).

### Branching and Annihilating Random Walks Reproduce the Dynamics of Mammary Morphogenesis

**Figure 3:**
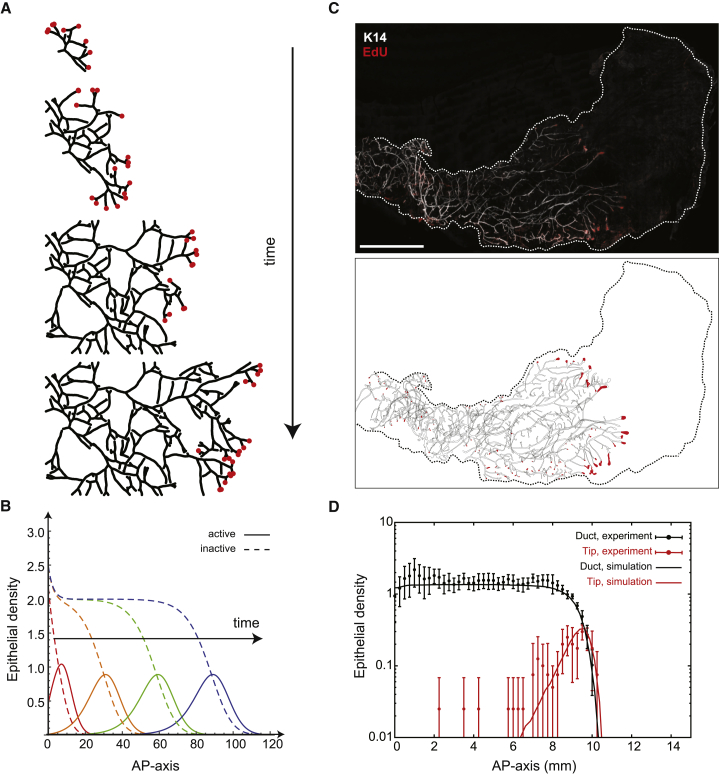
Branching and Annihilating Random Walks Reproduce the Kinetics of Mammary Invasion (A) Numerical simulation of the model at different developmental time points with ducts shown in black and active tips in red. (B) Theory predicts a self-organized solitary pulse of active tips positioned at the growing edge of the network, leaving behind a trail of inactive ducts of constant density. (C) 3D reconstruction of a fourth mammary gland following an EdU pulse at 5 weeks showing the position of active tips. Active tips are localized preferentially at the invasion front, mirroring qualitatively the prediction of the model. (D) Density profiles of ducts (black) and fully proliferative tips (red), averaged over n = 4 glands, alongside theory (red and black lines, respectively) revealing good quantitative agreement. Error bars represent mean and SEM. Scale bar, 5 mm. See also Figure S3.

Having established how the final state of the mammary epithelium is specified, we turned to examine whether the full dynamics of growth could also be predicted quantitatively. To gain insight into the nature and parametric dependences of the growth dynamics, we considered the hydrodynamic limit of the model in which the kinetics is captured by a mean-field theory, a manifestation of a “two-species Fisher-KPP equation” (Fisher, 1937) (Method Details):

(1) $$\{\begin{array}{c} \partial_{t}a=D\nabla^{2}a+r_{\text{b}}a\left(1-\frac{a+i}{n_{0}}\right)\\ \partial_{t}i=r_{\text{e}}a+\frac{r_{\text{b}}}{n_{0}}a\left(a+i\right) \end{array},$$

where *a*(*x*,*t*) and *i*(*x*,*t*) denote, respectively, the local concentration of active (tip) and inactive (duct) segments or “particles.” Referring to the description of the model dynamics above, active particles diffuse with diffusion constant *D* while producing inactive segments at rate *r*_e_ (reflecting the process of ductal elongation), branch at rate *r*_b_, and annihilate when they meet another particle (reflecting the process of tip inactivation), giving rise to a logistic growth term saturating at a total steady-state density, *n*_0_ (for details of how Equation 1 emerges from the stochastic model and can be related to biological signaling pathways, see Method Details). Within this framework, both theory and numerical simulations predict that, during expansion, active tips become self-organized into a narrow pulse at the growing front of the developing epithelium, traveling at constant speed as a solitary wave and leaving in its wake an inactive ductal network of constant density (Figures 3A, 3B, and S4D–S4I; Movie S1).

From a biological perspective, this behavior provides a natural explanation for the constant speed of invasion, a robust feature of mammary morphogenesis (Paine et al., 2016). At the same time, the theory predicts that ducts should be spatially patterned at a constant density (Figures 1A and 1B), while active tips should localize in a predictable pulse-shape distribution at the edge of the invading front. To test these predictions quantitatively, we performed EdU-pulse labeling of mice at 5 weeks of age (approximately the mid-point of branching morphogenesis of the mammary gland) and used whole gland reconstruction to both quantify the morphology of the network (Figures 3C and S4J) and define the regional localization of active tips (defined as proliferative tips with >50% of EdU^+^ cells, Figures S4J and S4K). Importantly, we found good qualitative agreement between experiment and theory, with active tips present at the edge of the growing front and a remarkably constant density of trailing ducts (Figure 3D). Quantitatively, analysis of the spatial profile at the growing front showed that the density of active tips decayed exponentially both ahead and behind the front, with the decay length of the former larger than the latter by a factor of $\left(\sqrt{2}-1\right)$, all key and non-trivial predictions of the Fisher-KPP dynamics (Figures S4F–S4I; Method Details).

Together, these results suggest that the global spatiotemporal dynamics of mammary ductal morphogenesis can be understood as a process of self-organization following from a program of stochastic tip bifurcation arrested by tip termination at the intersection with neighboring ducts.

### Giant Density Fluctuations and Self-Organized Directional Invasion during Mammary Morphogenesis

**Figure S5:**
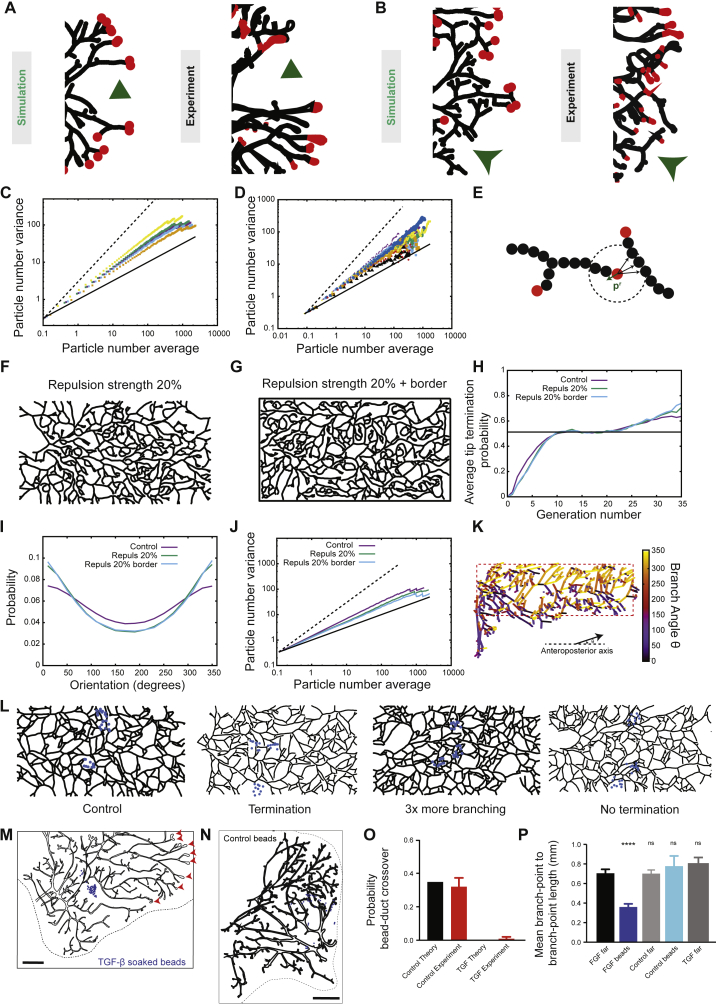
Density Fluctuations and Ordering in Branching and Annihilating Random Walks, Related to Figures 4 and 5 (A and B) Comparison between representative experimental tip configuration at the edge of the invasion front and numerical simulation reveals large tip density fluctuations due to stochastic random exploration of space (arrowhead, [A], zoom from Figure 3C) or stochastic massive tip termination (arrow, [B], from the third mammary gland). (C) Theoretical particle number variation versus average in log-log plot, for increasing branching probability *r*_*b*_, showing giant number fluctuations. Increasing *r*_*b*_ (i.e., going further from the critical point) produces lower exponents α of giant number fluctuations. Thick and dashed black lines represent resp. power laws of exponent 0.5 and 1. We show respectively *r*_*b*_ = 0.05 (α = 0.74, yellow), *r*_*b*_ = 0.85 (α = 0.69, green), *r*_*b*_ = 0.1 (control value, α = 0.67, purple), *r*_*b*_ = 0.12 (α = 0.66, blue) and *r*_*b*_ = 0.2 (α = 0.62, orange). (D) Experimental particle number variation versus average in log-log plot, for each of the n = 14 mammary glands analyzed. Although some level of dispersion exists, all glands are robustly showing giant number fluctuations. The thick black line represents a power 1/2, indicative of equilibrium, while the dashed black line represents a power 1. (E) Schematic of the repulsion implementation. Active tips sense neighbors over a radius of *R*_*r*_ around them, and in addition for their usual displacement of *l* in a direction **p**, make a step of *f*_r_ in the direction -**p**^*r*^ (green arrow), **p**^*r*^ being a unit vector which averages over all tip-neighbor vectors. (F–J) Sensitivity of the results of the model as a function of the repulsion strength *f*_r_. We perform simulations with a small strength (green lines, *f*_r_ = 0.2, F) for the repulsion between tips and ducts only, as well as simulations with the same strength (blue lines, *f*_r_ = 0.2, G), but where tips are also repulsed by the borders of the fat pad (drawn in black). We compare in each case the termination probability (H), the distribution of branch angles (I), as well as the exponent of giant number fluctuations (J, thick and dashed black lines represent resp. power laws of exponent 1/2 and 1). Increasing the repulsion strength enhances directionality and decreases the spatial density fluctuations (as expected for such a repulsive mechanism). However, the convergence toward balance between termination and branching probabilities is largely unaffected by local repulsion in two dimensions. (K) Color-coded branch angle distribution in a fourth mammary gland at 8w (same of Figure 1A, see also Figure 4C). (L) Simulations of bead experiments with various assumptions (left to right): control beads, beads provoking termination, beads promoting branching and beads inhibiting termination. (M) Additional typical example of a reconstructed third mammary gland in the presence of TGF-*β1* soaked beads (blue spheres, same concentration as Figure 5A). Active TEBs are marked by red arrowheads. (N) Additional typical example of a reconstructed third mammary gland in the presence of control, PBS with 0.1% BSA soaked beads (blue spheres). (O) Quantification of bead-duct cross-over in the presence of control versus TGF-β1 soaked beads, compared to its theoretical counterpart from (L). (P) Quantification of average branchpoint to next branchpoint distance in the presence of control versus FGF10 soaked beads, in regions close versus far to the beads, as well as far from TGF-β1 soaked beads. This displays a specific increase of branching rate close to the FGF10 beads. Error bars represent mean and s.e.m. Scale bars: 2 mm.

Although the proposed mechanism of branching morphogenesis can ensure a uniform density of ducts, statistical fluctuations during growth generate large spatial variations in the distribution of active EdU^+^ tips (Figure 3C). Indeed, the EdU-pulse assay reveals duct-depleted regions formed either by chance mass termination of tips (Figure S5A) or locally “divergent” flows of active tips randomly exploring other regions (Figure S5B), both behaviors being well-reproduced in the numerical simulations of the model dynamics. Importantly, according to the rules of the model dynamics, the trailing distribution of newly formed ducts is frozen or “quenched” in the fat pad. Therefore, we expect that the statistical fluctuations of epithelial density should persist in the mature network. Thus, in addition to the prediction of the average density profiles of active tips and mature ducts, the model makes further key quantitative predictions on the statistical properties of spatial density fluctuations.

**Figure 4:**
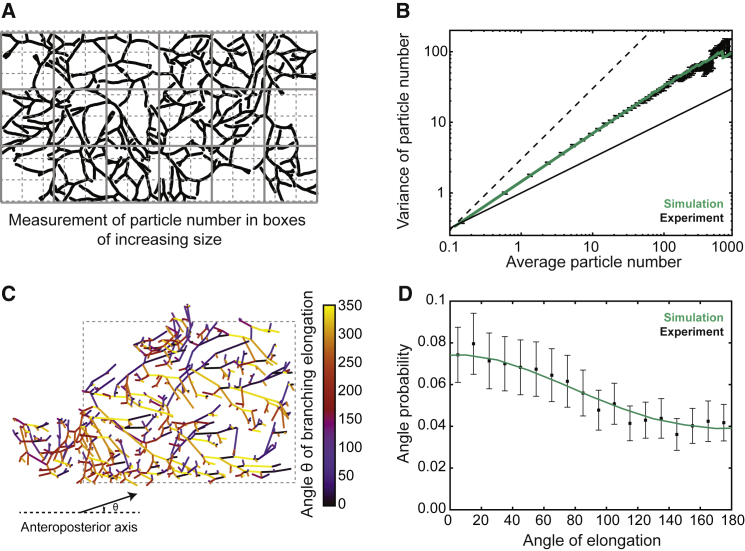
Self-Organized Properties of BARWs Predict Both Giant Density Fluctuations and Emergent Directional Bias of Ducts (A and B) Experimental variance (y axis) versus average (x axis) of duct volume in boxes of increasing size, *L* (A). The variance in density of the gland at different length scales grows as a power law (B, black bars), with an exponent larger than 1/2 (B, thin and dashed black lines represent exponents of 1/2 and 1, respectively), indicative of giant number fluctuations, and quantitatively predicted by the BARW model (green line). (C and D) Self-organized directional invasion proceeds from local negative interactions. (C) Representative example of the outline of an 8-week fourth mammary gland (same as Figure 2B), where the angle θ of each branch segment is calculated relative to the AP-axis. (D) Experimental (black bars) and theoretical (green line) distributions showing probabilities of finding a branch growing with a given angle θ. The experimental distribution is predicted quantitatively by the model even in the absence of a large-scale directional gradient. Error bars indicate mean and SEM. See also Figures S4 and S5.

We thus quantified these fluctuations by defining the spatial average, *n*_L_, and SD, (Δ*n*)_L_, of duct volume in boxes of viable size *L* (see Figure 4A for a schematic). For systems at equilibrium (in which each elemental process is equilibrated by its reverse, the property of detailed balance), the central limit theorem requires that ${\left(\Delta n\right)}_{L}=n_{L}^{\alpha }$ with the exponent α = 1/2. By contrast, in systems characterized by non-equilibrium fluctuations, α can take values larger than 1/2 (Ramaswamy et al., 2003, Narayan et al., 2007)—the phenomenon of giant number fluctuations. Indeed, using the same parameter set as before, model simulations revealed a robust power law dependence of (Δ*n*)_L_ (Figure 4B, green line), with an exponent α_theory_ ≈ 0.66, that increased with decreasing branching rate (Figure S5C).

Turning to previous mammary gland reconstructions at 8 weeks of age, we found many instances of large spatial density fluctuations that could not be accounted for by boundary effects, or by the presence of obstacles such as lymph nodes. We therefore applied the same statistical approach to determine experimentally the quantitative dependence of (Δ*n*)_L_ and *n*_L_ (n = 14 glands from 7 mice). Strikingly, this analysis revealed a robust power law dependence over more than three orders of magnitude (black dots, Figure 4B), with an exponent of α_exp._ ≈ 0.65 ± 0.02 (mean ± SEM), consistent with giant number fluctuations *in vivo*. Moreover, the experimental data collapsed on the theoretical curve with extremely high precision (Figures 4B and S5D), emphasizing the robustness of the model prediction (*R*^2^ = 0.90, $R_{\text{log}}^{2}=0.99$). Overall, this analysis uncovers an unexpected out-of-equilibrium feature of branching morphogenesis *in vivo* and serves as a strong test of the validity and predictive power of the BARW model. In particular, this shows that, while the proposed mechanism enforces (in a self-organized manner) a robust and constant averaged epithelial density, the local density is, as a result, only weakly regulated.

A further ubiquitous feature of mammary gland morphogenesis is the appearance of directional biases in the growth of the ductal network, suggestive of a mechanism that guides tips distally (Figures S5E–S5J). Indeed, quantification of the distribution of angles θ between a given branch and the horizontal proximal-distal axis (Figures 4C and S5K) revealed a 2-fold bias toward a proximal-to-distal orientation (Figure 4D). A puzzle in the field has been the lack of identification of any large-scale gradient that could cause this anisotropy (Gjorevski and Nelson, 2011). However, we reasoned that such a directional bias could derive naturally from the BARW model, even in the absence of global chemical cues or gradients, because branches growing toward the proximal region are more likely to terminate against existing ducts (i.e., less likely to give rise to progeny), resulting in an “effective” and self-organized bias emerging from isotropic short-range interactions. To test this hypothesis, we computed the theoretical prediction from the same model as above. Strikingly, the model was able to predict quantitatively the experimental profile (*R*^2^ = 0.95, Figure 4D), suggesting that directional bias may simply emerge as a natural consequence of the BARW model.

### Molecular Basis of Tip Termination and Branching

**Figure 5:**
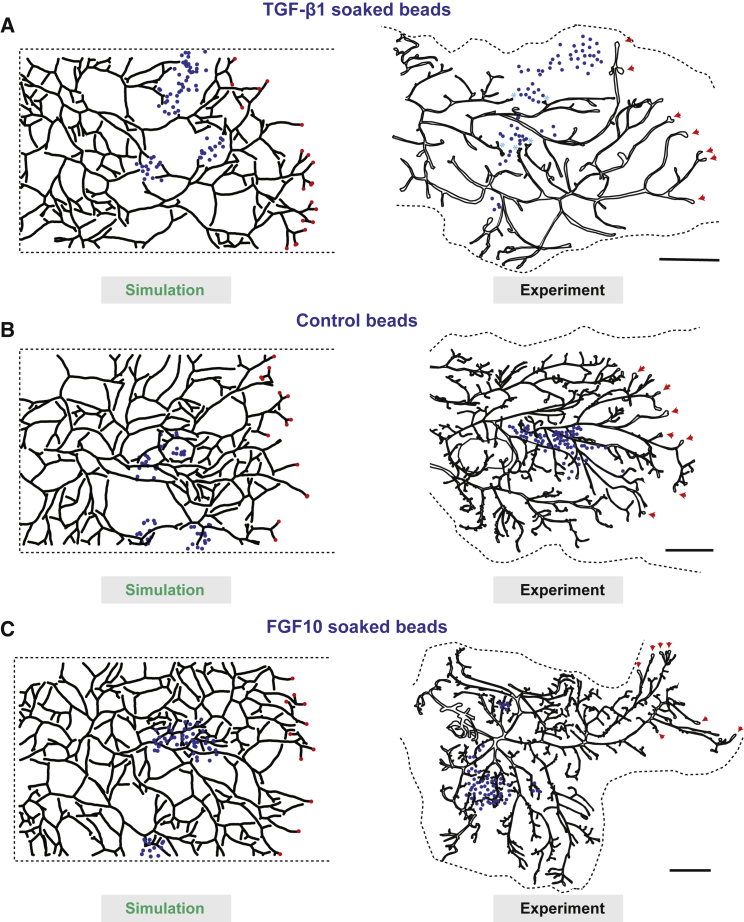
Perturbation Experiments Reveal the Molecular Basis of Termination and Branching (A) Comparison between a representative reconstructed fourth mammary gland (right) in the presence of TGF-β1 soaked beads (blue spheres) and a simulated theoretical counterpart (left). This confirms tip termination in proximity (light blue asterisks) to TGF-β1 soaked beads. (B) Comparison between a representative reconstructed fourth mammary gland (right) in the presence of control inactive beads soaked in PBS with 0.1% BSA (blue spheres) and a simulated theoretical counterpart (left). (C) Comparison between a representative reconstructed third mammary gland (left) in the presence of FGF10 soaked beads (blue spheres) and a simulated theoretical counterpart with local 2-fold increase in branching rate (right). Active TEBs are marked by red arrowheads. Scale bar, 2 mm. See also Figure S5.

Finally, given the importance of tip annihilation in our framework, we sought to test in a more direct way its underlying molecular basis. Ectopic delivery of TGF-β by large pellets has been shown to reversibly inhibit mammary ductal growth (Silberstein and Daniel, 1987). Therefore, to test the local action of TGF-β signaling, we implanted small TGF-β1-soaked agarose beads into the mammary fat pads of 4-week-old mice and waited for 2 weeks before sacrificing the mice (Figure 5A; Method Details). Importantly, as predicted by the theoretical simulations (Figures 5A, S5L, and S5M; Method Details) and as opposed to experiments with control beads soaked in PBS with 0.1% BSA (Figures 5B, S5N, and S5O), we found that mammary ducts never colonized regions rich in TGF-β1 beads, while we could observe numerous events of tips having stopped in their close proximity (100–200 μm, blue asterisks on Figure 5A). By contrast, the branching pattern was unaffected in regions devoid of beads (Figures 5A and S5P). These observations support the hypothesis that chemical signaling from maturing ducts regulate the termination of active terminal end-buds and implicate a role for TGF-β1 in providing the cue in a very local manner.

Next, we wished to assess quantitatively the effect of known positive regulators of branching morphogenesis. We thus performed a similar assay using FGF10-soaked beads (Figure 5C), as FGF10 has been identified as the predominant stromal FGF ligand expressed during pubertal mammary morphogenesis (Zhang et al., 2014). Notably, we found that FGF10 induced a 2-fold increase in branching, consistent with its proposed role in driving branch initiation in *in vitro* studies (Zhang et al., 2014), with a corresponding densification of the network close to beads, which was well-reproduced in model simulations (see Figure 5C; Method Details).

Together, these two sets of experiments provide both an additional test of the branching and annihilating random walk framework and a molecular basis for the regulatory program.

### Kidney Morphogenesis as a 3D Branching-Annihilating Random Walk

**Figure 6:**
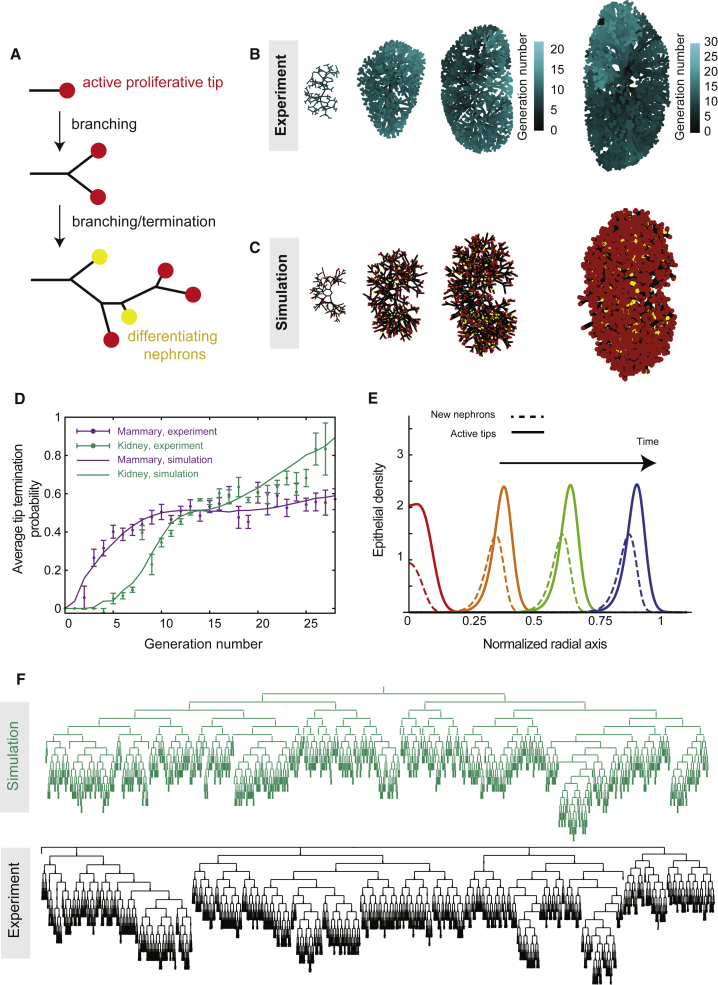
Branching and Annihilating Random Walks Can Reproduce Quantitatively the 3D Kidney Topology (A) Schematic of kidney morphogenesis as a stochastic branching process where active tips (red) either elongate, branch, or stop contributing to branching via nephron differentiation (yellow). (B) Reconstructions of murine kidney at E13, E15, E16, and E18 (left to right) with generation number of segments color coded in blue. (C) Typical output of numerical simulations of BARW model at corresponding time points. (D) Experimental versus theoretical tip termination probability as a function of generation for mammary gland (purple) and kidney (green), using the radius of termination *R*′_a_ = 0.25 as the only fitting parameter. (E) The model predicts a self-organized zone of active tips growing at the periphery of the kidney, followed spatially by a domain of tip termination, reminiscent of the nephrogenic zone observed *in vivo*. (F) Tree representation of a E17 kidney branching topology (top, green) and the output of the theory at the corresponding time point (bottom, black). Error bars represent mean and SEM. See also Figure S6 and Movie S2.

So far, we have restricted our analysis to the quasi 2D geometry of the mouse mammary gland. Therefore, to address the potential generality of the model to other organs, we considered the 3D incarnation of the BARW model using the development of kidney as a 3D system. During kidney morphogenesis, the ureteric bud, a single outgrowth that arises around embryonic day 11 (E11) from the nephric duct, arborizes to form the collecting system through a repeating process of mainly dichotomous branching. During the course of this iterative branching process, tips induce an aggregate of adjacent cap mesenchyme to undergo a mesenchymal to epithelial transition, thereby initiating the first steps of nephrogenesis, i.e., the formation of nephrons, the kidney’s filtration unit. These aggregates continue to mature while the renal connecting tubule concomitantly forms and joins these nascent nephrons with branching collecting ducts (Short et al., 2014, Sampogna et al., 2015, Cebrián et al., 2004). Crucially, as kidney development progresses, a growing subset of older ureteric tips continue to fuse with adjacent maturing nephrons. Once occupied, these tips are thought to no longer contribute to further branching (Sampogna et al., 2015, Costantini and Kopan, 2010) so that they can be considered to have undergone branching termination (Figure 6A).

**Figure S6:**
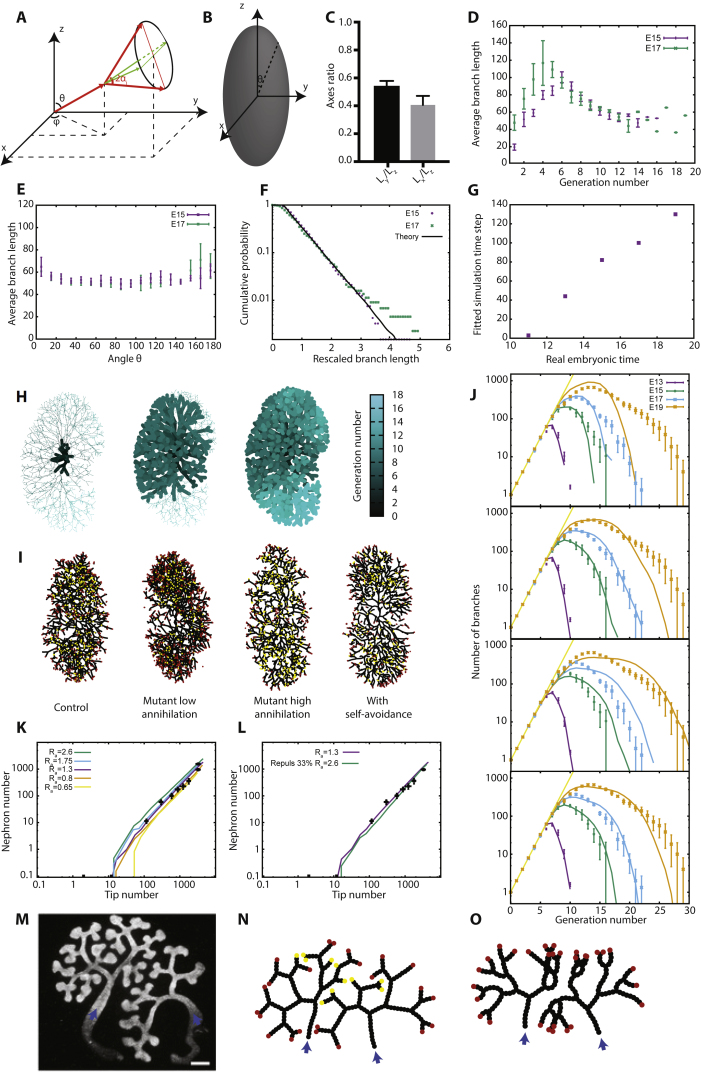
Branching and Annihilating Random Walks in a 3D Geometry, Related to Figure 6 (A) Definition of the angles θ and φ in spherical geometry, as well as the geometry of branching events. The two branches produced upon branching make an angle of *α*_*i*_ with the original branch, and are diametrically opposite to each other, while being free to choose the plane of bifurcation randomly. (B) Schematics of the definition of long axis (z), medium axis (y) and short axis (x) to align experimental reconstructions of kidneys, as well as definition of the angle θ of a given branch in the kidney used in (E). (C) Experimentally measured ratio between the long, medium and short axes of the kidney (n = 5 kidneys averaged from E16 to E19). (D) Average branch length as a function of the branch generation number for E15 and E17 kidneys (n = 3 each). (E) Average branch length as a function of the angle θ for E15 and E17 kidneys (n = 3 each), showing only a weak dependency (branch generations *>* 10 included). (F) Cumulative distribution of branch length (rescaled by its average) for E15 and E17 kidneys (n = 3 each, branch generations *>* 10 included), which is very well-fitted by an exponential distribution with a refractory period (cut-off below which branching cannot occur). (G) Correspondence between the embryonic time of segmented kidneys and the best-fit value for the simulated time step (from the total number of branches at that given time step), used to fix the timing of the theoretical curves from Figures 7A, 7C, and 7D. (H) Three-dimensional reconstructions of a wild-type E15 kidney, with branch generation number color-coded in shades of blue. We show successively only the first 5 branching levels (left), the first 10 branching levels (middle), and the full kidney (right), to emphasize the larger number of branching levels along the long axis *z* of the kidney. (I) Sections of typical simulation outputs of full three-dimensional simulations of kidney, for fours conditions, from left to right: section from a simulation using the default values used to fit wild-type kidneys (from Figure 7), i.e., with an annihilation radius of *R*^*’*^_a_*= 0.25*, section from simulations of mutant reducing the annihilation radius by half (*R*^’^_a_*= 0.125*), section from simulations of mutants increasing the annihilation radius two-fold (*R*^*’*^_a_*= 0.5)* and section from simulations adding self-avoidance properties (strength *f*_r_*= 0.33*, *R*^*’*^_a_*= 0.5),* showing a more ordered branch spatial distribution. In all cases, one observes the self-organization of a noisy front of active tips at the edge of the kidney, which becomes wider with decreasing annihilation radii. (J) Experimental versus theoretical average number of branches per generation as a function of time (E13 in purple, E15 in green, E17 in blue, and E19 in orange, n = 3 kidneys each), for several variations of the experimental parameters. Top panel: Theoretical branch number distributions without any annihilation (*R*_a_ = 0), showing that the variability in branch number per generation does not arise solely from a pure stochastic branching process. Middle-top panel: Theoretical branch number distributions for a lower annihilation radius halved compared to control (*R*^′^_a_ = 0.125), showing an underestimation of heterogeneity at E19. Middle-lower panel: Theoretical branch number distributions for a higher annihilation radius doubled compared to control (*R*^′^_a_ = 0.5), showing an overestimation of the heterogeneity at E19. (K and L) Experimental (n = 3 kidneys for each time point) versus theoretical number of inactive particle (i.e., nephrons, measured via glomeruli numbers experimentally) as a function of tip (active of inactive) number (black squares). We show on (K) the theoretical curves for decreasing annihilation radii: *R*^′^_a_ = 0.5 (green), *R*^′^_a_ = 0.33 (blue), *R*^′^_a_ = 0.25 (default, purple), *R*^′^_a_ = 0.15 (orange), *R*^′^_a_ = 0.125 (yellow) and on (L) the theoretical curves for default parameter (*R*^′^_a_ = 0.25, purple) without self-avoidance, with avoidance, showing an underestimation of the nephron number (*R*^′^_a_ = 0.25, *f*_r_ = 0.33, green) and with avoidance together with a larger self-annihilating radius (*R*^′^_a_ = 0.5, *f*_r_ = 0.33, blue). Error bars represent mean and s.e.m. (M) Snapshot, reproduced from (Davies et al., 2014), of a culture experiment where two intact kidneys were grown in proximity (blue arrows). (N) Simulation of (M) in the case of pure termination without repulsion. (O) Simulation of (M) in the case of pure repulsion without termination. Scale bar: 100 μm.

Motivated by these findings, we thus considered whether the BARW model could predict kidney morphogenesis. Indeed, the convergence of the BARW model toward balanced ductal bifurcation and termination described above is quite general, applying in all dimensions. However, simulating the model dynamics in 3D (Figures 6B, 6C, and S6) revealed that this convergence, for the same annihilation radius, occurs on much longer timescale (around 3 versus 10 generations, on average, Figure 6D). This behavior can be explained intuitively through differences in the frequency of random collisions between ducts and tips, which become much rarer in 3D as compared to 2D. From a biological perspective, this would mean that the topology of 3D branched organs should appear to be predominantly geometric (deterministic) early in development, displaying serial rounds of symmetric branching events without termination and only later becoming stochastic in character. Interestingly, such behavior is qualitatively consistent with recent reports by several groups using detailed 3D reconstructions (Short et al., 2014, Sampogna et al., 2015), showing structural heterogeneity only at higher branch levels. We therefore analyzed original and more recent data from (Sampogna et al., 2015), involving kidney reconstructions from E12 to E19, to test whether the same framework could apply during the seemingly non-stereotypical later phase of morphogenesis (Figures 6C and S6).

To develop a more precise quantitative comparison, we considered a numerical simulation of the branching dynamics in an unconfined 3D geometry (Figures S6A–S6C; Movie S2). In this case, the dynamics depends only on the ratio of the annihilation radius to the characteristic duct length, $R_{\text{a}}^{\prime }=R_{\text{a}}/l_{\text{d}}$ (Figures S6D–S6F). In contrast to the 2D setting, this parameter becomes crucial in 3D, where the probability of two branches to cross becomes of measure zero. Moreover, as kidney expands anisotropically, we renormalized all rate constants with respect to growth orientation to match the experimental aspect ratio (Figure S6C; Method Details).

Interestingly, with $R_{\text{a}}^{\prime }=0.25$, a value close to that found for mouse mammary gland, we could reproduce with high precision the growth characteristics of the E19 mouse kidney, as exemplified by the evolution of the tip branching versus termination probability as a function of branch level (Figure 6D). As mentioned above, ductal evolution is characterized by a protracted early phase of symmetric branching, converging slowly toward balanced fate. $R_{\text{a}}^{\prime }$ is thus the key and only fitting parameter, and all subsequent comparisons to experiments do not involve the adjustment of any additional parameter.

**Figure 7:**
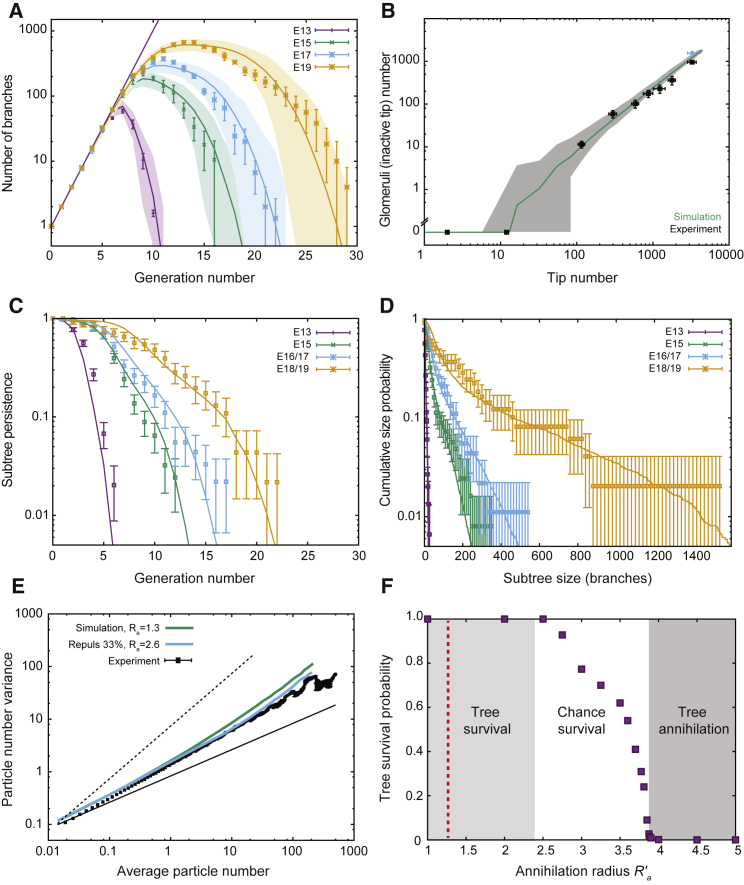
Branching and Annihilating Random Walks Can Reproduce Quantitatively the Detailed Properties of Kidney (A) Using the radius of termination *R*_a_ as the only free parameter, the model predicts well the number of segments per generation at different time points of embryo development (E13, E15, E17, and E19 in purple, green, blue, and orange, respectively). (B) Inactive tip number (assessed indirectly via glomeruli staining from [Sampogna et al., 2015] in black, or glomeruli counting via a method of acid maturation from [Cebrian et al., 2014] in blue) versus total number of tips, displaying a power law after a phase of purely symmetric branching, predicted by the model (green). (C) Subtree persistence at different developmental time points (squares) compared to the model (lines). (D) Cumulative subtree size distribution at different developmental time points (squares) compared to the model (lines). (E) Variance (y axis) versus the average (x axis) duct volume in a box of size *L* (experiments in black) in kidney, showing an exponent larger than 1/2 (thin and dashed black line represent exponents of 1/2 and 1, respectively), indicative of giant number fluctuations. The green and blue lines are predictions from default model (no repulsion, *R*′_a_ = 0.25), and model with repulsion (*f*_r_ = 0.33, *R*′_a_ = 0.5). (F) Tree survival probability versus termination radius, showing a phase transition above which kidney systematically become fully annihilated. Red dashed line shows the best-fit value of *R*_a_ used in (A)–(D). Shaded areas represent 95% confidence intervals, and error bars represent mean and SEM. Lines in (A)–(D) are model predictions, using the parameter *R′*_a_ fitted from Figure 6D. See also Figure S7 and Movies S2 and S3.

**Figure S7:**
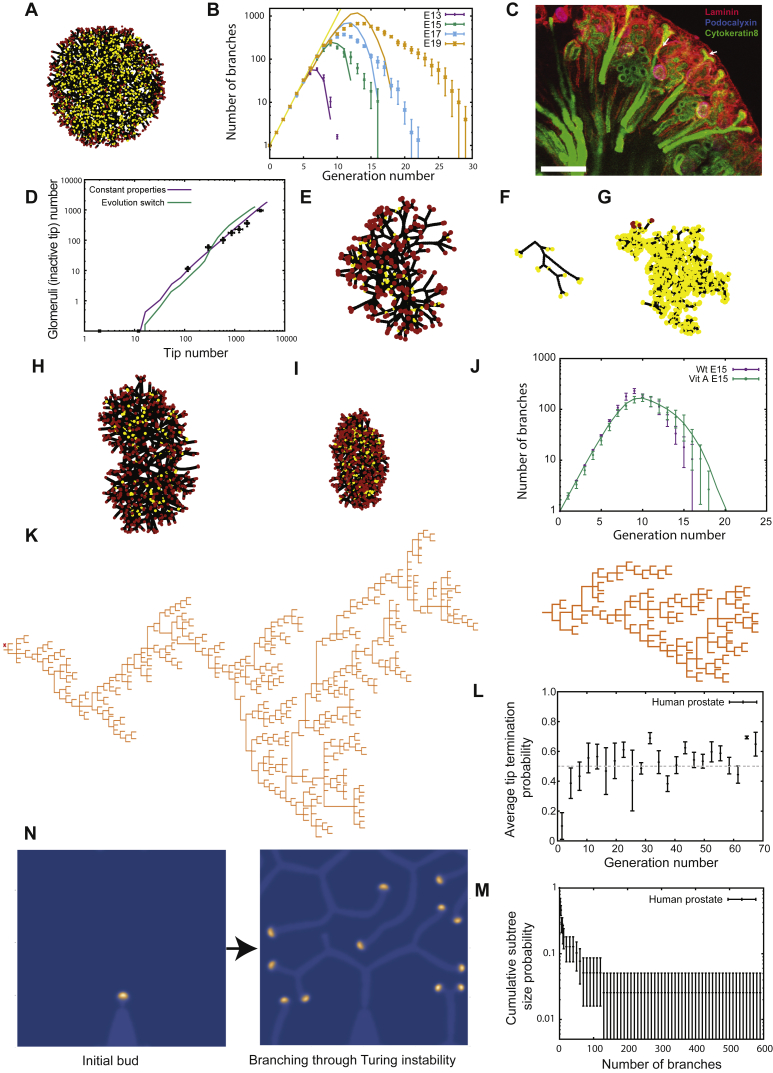
Branching and Annihilating Random Walks as a Generic Framework to Understand Pathologies and Other Branched Organs, Related to Figures 6 and 7 (A and B) Simulations using control parameters, but in an anisotropic setting lead to a similar phenomenology of a self-organized pulse of active tips at the edge (section of a simulated tree shown on [A]), although with decreased tree heterogeneity ([B], n = 3 kidneys for each time point). (C) Section of a E17 murine kidney, displaying collecting epithelial ducts (green, stained for cytokeratin 8) and a nephrogenic zone positioned at the growing periphery of the tissue: glomeruli (used as a proxy for maturing nephrons) stain with both the red (laminin, renal basement membrane) and blue (podocalyxin for podocytes in the glomerulus) channels and appear pink. Bifurcating tips (white arrows) can also be observed at the tissue periphery. (D) Theoretical number of inactive particle (i.e., nephrons, measured via glomeruli numbers experimentally) as a function of tip (active of inactive) number (data: black squares), both for the control model described in the main text with time-invariant parameters (purple line), and for time-varying parameters (with *R*^′^_a_ = 0.125 before E15 and *R*^′^_a_ = 0.5 afterward), which an absence of power-law scaling (green curve). (E) Typical output of a numerical simulation from the phase diagram of Figure 7F, for an annihilating radius *R*_a_ = 2.5, with systematic tree survival through an initial excess of branching compared to termination. (F and G) Two typical outputs of numerical simulations from the phase diagram of Figure 7F, for an annihilating radius *R*_*a*_ = 3.9, i.e., close to the critical point. Simulated trees then stochastically self-annihilate at varying sizes, from rudimentary trees [F]) to complex structures (G). (H and I) Two typical outputs of numerical simulations of a E15 kidney, using the default model parameter (H) and an averaged branch length halved (I), mirroring Vitamin-A deficient kidneys. (J) Experimental versus theoretical average number of branches per generation in E15 kidneys for wild-type (purple) and Vitamin-A deficient mice (green, data from Sampogna et al., 2015), along side the theoretical predictions from the simulations shown in (H) and (I). (K) Two examples of tree topology from the three-dimensional reconstruction of a subunit of human prostate, displaying heterogeneity and numerous early termination events. (L) Tip termination probability as a function of generation, for n = 5 human prostates, showing a rapid convergence toward a balance between tip termination and tip branching (green horizontal line). (M) Subtree (defined as starting at generation 6) heterogeneity, assessed via its cumulative size distribution. (N) Numerical simulation of a four species Turing-Meinhart system (see STAR Methods for details and parameters). We represent a density plot of the concentration of the activator *a*, which localizes at the growing tip. High concentrations are color-coded in yellow and low concentration in blue. The system performs branching and annihilating persistent random walks, and could therefore serve as a molecular basis for our model. Error bars in (B) and (H) represent mean and s.e.m and in (I) a confidence interval of 1 s.d. In (A) and (E)–(I), active tips are red, inactive tips yellow, and ducts black. Scale bars: 200 μm.

We then compared the experimental and theoretical topologies of kidneys (Figure 6F), as well as the distributions of branch number as a function of branch level across a wide range of developmental time points (Figures 7A, S6H, and S6I). Given the simplicity of the model, these results showed remarkably good correspondence, revealing an initial phase of geometric ductal expansion (with the number of branches at level *n* growing as 2^*n*^), followed by a plateauing and widening of the distributions, a manifestation of increasing ductal termination (*R*^2^ = 0.93 at E13, *R*^2^ = 0.95 at E15, *R*^2^ = 0.94 at E17, and *R*^2^ 0.93 at E19, Figures S6I–S6L). Importantly, this behavior does not arise from purely geometric anisotropies, as can be seen in the same simulation without termination events $\left(R_{\text{a}}^{\prime }=0\right)$ (Figure S6J), or with termination in an isotropic geometry (Figures S7A and S7B).

### Stochasticity in Kidney Morphogenesis and Nephron Number Specification

In common with the mammary epithelium, growing tips were also predicted to become self-organized into a pulse of activity at the periphery of the developing kidney, while newly formed nephrons were predicted to form as a secondary pulse behind this front (Figure 6E). Such behavior matched the known organization of the kidney into a nephrogenic zone positioned at the growing periphery of the tissue (Figure S7C) (Sampogna et al., 2015, Short et al., 2014). Moreover, although the time evolution of both the active tip and nephron density can depend on time variations in branching rate, plotting one versus the other provided a robust, time-independent test of model.

Using glomeruli, the capsule of capillaries located at the beginning of a nephron (Sampogna et al., 2015), as a proxy for the number of maturing nephrons, we found that, after an initial phase of pure tip production without any nephrons, both quantities robustly scaled experimentally, with the relationship well-fit by a power law (Figures 7B, S6K, and S6L). Such scaling argues for simple time-invariant rules underlying nephron specification, because deviations from this time-invariance would cause deviations from the observed scaling (as simulated in Figure S7D). Crucially, we then compared this observation to our model, using the same parameter set as that used above, and found a good prediction for the scaling relationship throughout the entire time course of embryonic development (*R*^2^ = 0.78, $R_{\text{log}}^{2}=0.97$).

To determine whether the model could also predict the detailed heterogeneity of branching structures as well as the averages, we examined the size distributions and persistence of subtrees at each time point (defined as in mammary gland). These were consistently broadly distributed, indicative of large-scale heterogeneity, and adopted similar functional dependences to their mammary counterparts, indicative of conserved (or universal) underlying properties. Crucially, the distributions of subtree persistence were consistently very well-fit by the model at all developmental time points (Figure 7C, *R*^2^ = 0.95 at E13, *R*^2^ = 0.99 at E15, *R*^2^ = 0.97 at E17, and *R*^2^ = 0.97 at E19) as were subtree size distributions (Figure 7D, *R*^2^ = 0.89 at E13, *R*^2^ = 0.99 at E15, *R*^2^ = 0.99 at E17, and *R*^2^ = 0.93 at E19).

Finally, although the model captures successfully several non-trivial features of the experimental data, arguing for conserved rules underlying both mammary gland and kidney morphogenesis, we noted that kidney reconstructions were characterized by a rather regular spacing between tips, which was consistently more ordered than the numerical simulations of the model (Figures S6H and S6I). Indeed, although we found again evidence of giant number fluctuations (Figure 7E), the model slightly overestimated the amplitude of fluctuations (Figure 7E), hinting that tips may be partially self-avoiding, as proposed by (Davies et al., 2014). To incorporate this effect into the model, we proposed that tips, in addition to their persistent random motion, are repulsed by neighboring tips and ducts when within a radius *R*_r_. With *v*_r_ defining the characteristic speed change induced by this repulsion, the ratio *f*_r_ = *v*_r_/*v* provides a measure of the strength of the repulsion (Quantification and Statistical Analysis). For large values of *f*_r_, termination becomes extremely rare, yielding a behavior inconsistent with the observed degree of kidney heterogeneity and nephrogenesis kinetics. However, for small values of *f*_r_ = 0.2 and a correspondingly larger value of the annihilation radius *R*_a_ (Quantification and Statistical Analysis), we could still obtain a satisfactory fit to the data (Figures S6J and S6L), while obtaining a more ordered kidney structure (Figure S6I). Interestingly, without further adjustment, this improved the fit to the observed density fluctuations (*R*^2^ = 0.75, $R_{\text{log}}^{2}=0.98$, Figure 7E).

These findings argue that, although self-avoidance is not the dominant characteristic of kidney morphogenesis (Figures S6M–S6O), it may cooperate with termination, i.e., nephron maturation, to produce a partially ordered structure. Notably, both elements of the model arise from purely local rules, maintaining the self-organizing character of branching morphogenesis. Indeed, this might explain why it can proceed robustly *in vitro* in the absence of external chemical or morphogen gradients (Davies et al., 2014).

### Branching Defects as a Route to Premature Termination of Branching Morphogenesis

Based on these findings, we then questioned their implications for pathologies of branched organs, such as kidney, which have been linked to defects in branching morphogenesis. For instance, hypertension has been proposed to be at least partially explained by insufficient nephron number (Brenner et al., 1988), whereas renal agenesis is a relatively frequent congenital defect in humans, mirroring the *GDNF* knockout in mice, that results in the formation of tiny rudimentary ductal trees in the kidney (Pichel et al., 1996).

Interestingly, by the stochastic nature of the BARW, chance events may also lead to the premature extinction of active tips, which annihilate against the existing ductal network, inhibiting kidney growth. Although the frequency of such events is negligibly small for the parameters of wild-type tissue (Figures 7F and S7E), higher values of the annihilation radius cause the extinction probability to increase dramatically (Figures 7F, S7F, and S7G; Movie S3). Numerical simulations reveal a critical point above which early extinction always occurs (Figure 7F; Quantification and Statistical Analysis), as well as a continuous transition to a non-zero extinction probability below this critical value, which could explain the variable nature of small ductal trees observed in *GDNF* knock-out mice.

Based on this insight, we examined branching morphogenesis on E15.5 littermate kidneys that developed under the condition of mild maternal-fetal Vitamin A-deficiency as described previously (Sampogna et al., 2015). These kidneys are nearly two times smaller than wild-type (Figures S7H and S7I) and display larger subtree size heterogeneity (n = 4 mice, p < 0.05), although the total number of branches remains normal (Sampogna et al., 2015). Noting that vitamin A-deficient kidneys were markedly smaller, we tested whether this measured decrease in branch length was enough to reproduce the enhanced heterogeneity, by producing earlier crowding-induced tip termination (Method Details). We found that a uniformly decreased branch length was indeed sufficient to reproduce quantitatively the changes in branch generation distribution in vitamin A-deficient mice (Figure S7J). Further studies will be needed to address, more generically, whether such mutant conditions can be understood in terms of ratio between branch length and termination radius, i.e., of their proximity to the annihilating critical point.

### Balance between Tip Termination and Branching Is Also Observed in Human Prostate

Finally, to further explore the generality of the proposed mechanism of branching morphogenesis, we turned to consider the human prostate, which consists of independent subunits branching independently from the urethra (McNeal, 1968). Organogenesis of the prostate shares key features of tip driven morphogenesis as described above in breast and kidney formation: the adult branching structure derives from epithelial ductal outgrowths into surrounding urogenital mesenchyme during embryogenesis and the immediate postnatal period (Powers and Marker, 2013).

From the tracing studies of ductal subtrees, based on large-scale 3D reconstructions of adult human prostates (n = 5 from 5 patients), some of which extended to 70 generations of branching, we found that some regions terminate early, with tips forming differentiated acini structures, while others grow extensively (Figure S7K). From a plot of the relative probability of tip termination versus branching, we found again that the overwhelming majority of subtrees displayed a striking degree of balance between ductal termination and branching (Figure S7L). Additionally, we found that the functional shape of the distribution was again similar to the other organs, with a few subtrees growing to up to 10 times the average subtree size (Figure S7M). These findings suggest that the paradigm uncovered for mammary gland and kidney morphogenesis may be translated to a priori different biological settings.

## Discussion

In this study, we have investigated how the branching pattern of the mouse mammary gland epithelium and kidney emerge throughout development. Using a combination of whole-organ 3D reconstruction, proliferation kinetics, and biophysical modeling, we have provided evidence that branching morphogenesis proceeds from the spatial competition of equipotent tips, which randomly explore space through a process of ductal elongation and stochastic branching. If this process occurred without competition between growing tips, branched organs would be characterized by stereotypical rounds of purely symmetric branching, with the number of branches increasing with branch level *n* as 2^*n*^. Indeed, such behavior would serve to minimize the time required to build a branched structure while filling space efficiently. However, reconstructions of mouse mammary gland, kidney, and human prostate reveal a different scenario, where tip terminations occur even at the earliest stages of branching morphogenesis and rapidly balance tip bifurcations at the population level.

Based on the scarcity of ductal crossovers in mammary gland, we propose that the dominant source of tip termination is the presence of neighboring ducts inhibiting growth. This provides a density-dependent feedback that naturally balances ductal branching and tip termination. This hypothesis challenges the concept of branching morphogenesis occurring through a rigid and deterministic sequence of genetically programmed events, and replaces it by a stochastic self-organizing model of development. After deducing the branching rate from *in vivo* measurements, our model predicts nearly perfectly and without adjustable parameters the network topology and spatial structures of adult mammary glands, while also making a number of additional non-trivial quantitative predictions.

In particular, it predicts the self-organization of active tips into a spatial domain or pulse, localized at the growing front of the network, which invades into the fat pad at constant speed, leaving behind a constant density of mature ducts. As a consequence, our model suggests that the directional invasion of the mammary gland toward the distal end of the fat pad does not need to be guided by a global chemotactic gradient, but instead can be explained quantitatively in a self-organized manner from the short-range annihilating properties of tips and ducts. As a non-equilibrium process, the BARW model predicts quantitatively the existence and scaling dependence of hallmark giant density fluctuations, which we verify experimentally.

Finally, we have shown that the model applies equally well in the 3D setting of the developing mouse kidney, reproducing accurately the network heterogeneity, with some subtrees colonizing large parts of the kidney while others terminate precipitously, as well as the spatiotemporal pattern of nephrogenesis. Such behavior suggests that this self-organized pattern of growth, consistent with the *in vitro* growth capability of kidney trees, may constitute a conserved (universal) mechanism of branching morphogenesis across different tissues, shifting the focus of future studies to the collective spatiotemporal fate control of branching and termination of entire tips, rather than on individual cells.

From a molecular mechanistic perspective, some of the processes underlying tip termination have been studied individually in several organs. In particular, inhibition of tip growth through TGF-β signaling has been demonstrated in both mammary and prostate glands (Silberstein, 2001, Powers and Marker, 2013). TGF-β is also a good candidate to provide crowding-induced feedback (Silberstein, 2001), as it is known to be diffusible in the stroma, is secreted by mature ducts, and has been shown both *in vitro* and *in vivo* to regulate the branching pattern of pubertal morphogenesis (Silberstein and Daniel, 1987), as confirmed here. Moreover, it was recently reported from *in vitro* culture experiments that the TGF-β superfamily, in particular Bmp7, was also implicated in crossover avoidance in kidney (Davies et al., 2014).

Thus, these findings question the underlying molecular basis of the BARW model. Given the diffusible nature of key underlying regulators, we investigated whether generic reaction-diffusion models could explain the BARW phenomenology. Interestingly, we found that such branching and annihilating dynamics can indeed emerge naturally and robustly from simple Turing-Meinhardt type models (Meinhardt, 1982, Guo et al., 2014) involving only spatial interactions of an activator, an inhibitor, and a consumed substrate (Figure S7N; Method Details). Taken as a whole, our study demonstrates that the morphogenesis of complex ductal tissues can be understood and predicted quantitatively on the basis of a remarkably simple set of local rules that direct the robust self-organization of a large-scale network structure.

## STAR★Methods

### Key Resources Table

**Table:** 

REAGENT or RESOURCE	SOURCE	IDENTIFIER
**Antibodies**		

rabbit anti-Keratin 14 Polyclonal	BioLegend	Cat# 905301 RRID: AB_2565048
rat anti-CD324 (E-cadherin) DECMA-1	Affymetrix eBioscience	Cat# 14-3249-82 RRID: AB_1210458
Alexa Fluor 488 donkey anti-rat IgG (H+L)	Thermo Fisher scientific	Cat# A21208 RRID: AB_2535794
Alexa Fluor 568 donkey anti-rabbit IgG (H+L)	Thermo Fisher scientific	Cat# A10042 RRID: AB_2534017

**Chemicals, Peptides, and Recombinant Proteins**		

Collagenase A	Roche Diagnostics	Cat# 10 103 578 001
Hyaluronidase	Sigma-Aldrich	Cat# H3884
DAPI solution (1mg/ml)	Thermo Fisher scientific	Cat# 62248
Vectashield Hard set	Vector Laboratories	Cat# H-1400
Recombinant Mouse FGF-10	R&D Systems	Cat# 6224-FG-025
Recombinant Mouse TGF-β1	R&D Systems	Cat# 7666-MB-005
Affi-Gel Blue Gel	Bio-rad	Cat# 1537301

**Critical Commercial Assays**		

Click-iT EdU Alexa Fluor 647 Imaging Kit	Thermo Fisher Scientific	Cat# C10340

**Experimental Models: Organisms/Strains**		

FVB/NJ	Taketo et al., 1991	https://www.jax.org/strain/001800
MMTV-Cre (FVB)	Wagner et al., 1997	https://www.jax.org/strain/003553
LSL-YFP (FVB)	N/A	https://www.jax.org/strain/006148

### Contact for Reagent and Resource Sharing

Further information and requests for resources, reagents and simulation tools should be directed to the Lead Contact, Prof. Benjamin Simons (bds10@cam.ac.uk).

### Experimental Model and Subject Details

#### Mice

All mice were female, bred on a FVB/NJ background, and in the age ranging from 3.5 – 8 weeks. Mice were randomly assigned to the experimental groups, and mice were not selectively excluded from the experiments. All animal procedures and experiments were performed in accordance with national animal welfare laws under a project license obtained from the Dutch Government, and were reviewed by the Animal Ethics Committee of the Royal Netherlands Academy of Arts and Sciences (KNAW). Mice were housed in a barrier facility in conventional cages and received food and water *ad libitum*. All personnel entering the barrier must wear protective clothing (including head caps, specials clogs). All animals were received directly from approved vendors or generated in house.

### Method Details

#### Mice Experimental Details

Mice of 3.5, 5 or 8 week-old were injected IP with 0.5mg 5-ethynyl-2′-deoxyuridine (EdU, Thermo Fisher Scientific) diluted in phosphate buffered saline (PBS). Mice were sacrificed 4 hours after EdU injection, and the 3^rd^, 4^th^, and 5^th^ mammary glands were collected and processed as whole-mount glands. For beads implantation experiments, 4 week-old MMTV-Cre/LSL-YFP mice were injected with 5 μl of Affi-gel blue beads coated with recombinant protein.

#### Local application of Affi-gel blue beads in mammary glands

Affi-gel blue beads (Bio-RAD Laboratories) were washed 3 times for 5 min in sterile PBS. Next, beads were incubated with excess of 50 μg/ml recombinant mouse TGF-β1 (R&D Systems) in 4mM HCL or 100 μg/ml recombinant mouse FGF-10 (R&D Systems) in PBS 0.1% BSA for 1 hour at 37°C to adhere the proteins to the beads. Control beads were incubated with PBS 0.1% BSA. Beads were pelleted after and resuspended in sterile PBS. To implant the beads, mice were anesthetized and a small incision was made to gain access to the 3^rd^ or 4^th^ mammary gland. A small pocket was made in the fat pad (so that they did not physically prevent the invasion of the mammary epithelium, but still delivered proteins in its vicinity) and 5 μl of beads was injected (approximately 30-60 beads per gland). After 2-3 weeks, mammary glands were dissected and processed as whole mounts.

#### Whole-mount immunofluorescence staining of mammary glands

Mammary glands were dissected and incubated in a mixture of collagenase I (1mg/ml, Roche Diagnostics) and hyaluronidase (50 μg/ml, Sigma-Aldrich) at 37°C for optical clearance, fixed in periodate-lysine-paraformaldehyde (PLP) buffer (1% paraformaldehyde (PFA, Electron Microscopy Science), 0.01M sodium periodate, 0.075M L-lysine and 0.0375M P-buffer (0.081M Na_2_HPO_4_ and 0.019M NaH_2_PO_4_) (pH 7.4)) for 2 hours at room temperature (RT), and incubated for 2 hours in blocking buffer containing 1% bovine serum albumin (Roche Diagnostics), 5% normal goat serum (Monosan) and 0.8% Triton X-100 (Sigma-Aldrich) in PBS. For EdU cell proliferation staining of whole-mount mammary glands, a click-it staining (Click-iT EdU, Thermo Fisher Scientific) was performed according to the manufacturer’s instructions before staining with primary antibodies as described above. Next, primary antibodies were diluted in blocking buffer and incubated overnight at RT. Secondary antibodies diluted in blocking buffer were incubated for at least 4 hours. Nuclei were stained with 4’,6-diamidino-2-phenylindole (DAPI, 0.1 μg/ml; Sigma-Aldrich) in PBS. Glands were washed with PBS and mounted on a microscopy slide with Vectashield hard set (H-1400, Vector Laboratories). Primary antibodies: anti-K14 (rabbit, Biolegend, 905301, 1:700) or anti-E-cadherin (rat, Affymetrix eBioscience, 14-3249-82, 1:700). Secondary antibodies: donkey anti-rat conjugated to Alexa 488 and donkey anti-rabbit conjugated to Alexa-568 (Thermo Fischer Scientific, A21208 and A10042 respectively, 1:400).

#### Whole-mount imaging of mammary glands

Imaging of whole-mount mammary glands was performed using a Leica TCS SP5 confocal microscope, equipped with a 405nm laser, an argon laser, a DPSS 561nm laser and a HeNe 633nm laser. All images were acquired with a 20x (HCX IRAPO N.A. 0.70 WD 0.5mm) dry objective using a Z-step size of 5μm (total Z stack around 200μm). All pictures were processed using ImageJ software (NIH, Bethesda, MD, www.nih.gov).

#### Models of branching and annihilating random walk

As a first attempt to model the dynamics, tip termination was first hypothesized to be mediated by contact between two actively proliferating tips. Such a model mapped directly onto the classical problem of branching and annihilating random walks. Classical branching and annihilating random walks (BARWs) are defined by a set of n walkers undergoing random walks in d spatial dimensions with a diffusion constant D. $r_{i}{\left(t\right)}_{i=1..n}$ denotes the position vector of each walker in space at a time t. In the general case, walkers A can either branch into m+1 new walkers:

(2) $$A\to \left(m+1\right)A,\;\text{at}\;\text{rate}\;r_{b},$$

or annihilate into an inert state I when two walkers meet locally in space,

(3) $$2A\to I,\;\text{at}\;\text{a}\;\text{rate}\;\text{that}\;\text{scales}\;\text{with}\;\text{volume}\;\text{as}\;r_{a}$$

with the branch multiplicity m being a strictly positive integer. Walkers that fall into the inactive state do so irreversibly and do not diffuse, nor interact with other walkers. The total number of walkers, n, active or inactive, therefore evolves in time. $s_{i}{\left(t\right)}_{i=1..n}$ denotes the state of each walker (with active denoted by 1 and inactive as 0). Defined in this form, this classical model has been studied extensively (Cardy and Täuber, 1996). Insight into the behavior of the model can be gained from the corresponding mean-field rate equation for the local density of walkers, n(t), which takes the form:

(4) $$\partial_{t}n\left(t\right)=r_{b}mn\left(t\right)-r_{a}n{\left(t\right)}^{2}$$

This equation predicts a pair of stationary solutions corresponding to an unstable inactive state (n=0) and a stable active state $\left(n_{s}=mr_{b}/r_{a}\right)$. However, when fluctuations around the mean-field approximation are taken into account, the situation is more complex: In particular, it has been shown that the system can belong to two distinct universality classes for either odd or even values of m. For even values of m, the parity of the number of active particles is conserved upon branching, falling into the parity-conserving class, while the case of odd values of m falls into the directed percolation universality class (Cardy and Täuber, 1996). Theoretical analysis of these two classes has been facilitated by the fact that they can be mapped on an Ising model, where branching and annihilation events can be mapped onto Glauber and spin-exchange Kawasaki dynamics (Odor, 2004).

In mammary gland and kidney, development is driven by proliferative tips or terminal end-buds (TEBs), which either advance forward, producing in their wake the ductal structure, or branch into two tips during a bifurcation event. In the model, the ductal region deposited by tips was thus considered inert, i.e., it did not proliferate further nor move. Moreover, most branching events are observed to involve tip bifurcation in which a single active tip divides into precisely two active tips. This translated to m=1 in the formalism above. Importantly, since the ductal trees constitute the past trajectories of each individual active walker (or tip), the entire time course of the process was reconstructed from the analysis of the final branching structure in space. Referring to the *in vivo* data (Figures 1A and S1), it was clear that the tip trajectories did not behave as temporally uncorrelated random walks but, instead, displayed a typical persistence length that could be read from the biological data. The model therefore had be amended by introducing a unit polarity vector, **p**_***i***_, which specified the direction of movement of a given walker. (The local velocity v of each walker was taken to be the same.) This polarity vector undergoes a persistent random walk with characteristic time $\tau_{p}$. Translated spatially, this implied a persistence length of ductal branches of $v\tau_{p}$ and the long-term (i.e., at timescales larger than $\tau_{p}$) diffusion constant of a walker of $v^{2}\tau_{p}$.

#### Mean-field dynamics of front invasion

To account for the spatial dynamics of branching morphogenesis, the mean-field theory above must be amended to take into account of the evolving spatial structure. In line with the biological system, the initial condition was taken as a single active walker positioned at one side of a finite domain (Figure S1 and Movie S1). Following classical results of simple branching processes (Fisher, 1937), the spatial dynamics in d -dimensions could be written as

(5) $$\{\begin{array}{l} \partial_{t}a=D\nabla^{2}a+r_{b}a\left(1-\frac{a}{a_{0}}\right)\\ \partial_{t}i=r_{e}a+\frac{r_{b}}{a_{0}}a^{2} \end{array}$$

where a(r,t) and i(r,t) represent, respectively, the local concentration of active and inactive particles and $\nabla^{2}$ denotes the Laplace operator. Related to the description above, active particles diffuse with a diffusion constant D, branch at a rate $r_{b}$ and annihilate when they meet, giving rise to a logistic growth term saturating at a density $a_{0}$. Inactive particles are constantly produced either when active particles move, or when two active particles meet. Their coefficient of diffusion is zero, expressing the fact that they remain immobile and inactive. The first of these Equations (5) translates to the well-known Fisher-KPP equation, after Kolmogorov-Petrovsky-Piscounov, which has been used as a simple approximation to describe front propagation (e.g., in the study of advantageous genetic mutations) (Fisher, 1937). The second equation, describing the concentration of inactive particles, is slave to the dynamics of the active particles, and does not provide any feedback. According to this dynamics, the theory thus predicted that active walkers would form a sharp front (of characteristic length $\sqrt{D/r_{b}}$), which invaded the tissue as a solitary wave, with velocity $V^{*}=2\sqrt{Dr_{b}}$. The associated wave profile links the unstable (a=0) solution at the growing front of the ductal network to the stable $\left(a=a_{0}\right)$ solution at the back (heteroclinic orbit).

Active tips thus formed a non-equilibrium stable steady-state (i.e., a constant density profile in time, but which was driven by constant and compensatory creation/annihilation events), which invades the unstable inactive state, a=0. However, this raised the problem of inactive particles that keep being produced at the back of the wave, driving a divergence in their concentration over time as $i\propto a_{0}\left(r_{e}+r_{b}\right)t$. As the wave propagated linearly in time, this translated into a linear concentration gradient toward the back of the waves, i.e., the concentration became higher and higher as x→∞ with slope $a_{0}\left(r_{e}+r_{b}\right)/V^{*}$ (Figure S4D). Such behavior was clearly unrealistic as a model of branching morphogenesis, calling for an alternative mechanism of active tip termination.

#### Revised model of tip termination

From the biological perspective, the regulation of tip termination could constitute an incredibly complicated process. For example, each walker/tip could be specified early in development with disparate properties, and programmed to terminate at a given time. At each bifurcation event, a given tip could segregate into two tips that both inherit a defined differentiation program. Alternatively, an extrinsic regulatory mechanism based, for example, on hormonal levels (Sternlicht, 2006) could operate, where tips would be allowed to grow and branch during a period T while they invade the full domain, becoming inactive on reception of a global signal at the end of this period. Such dynamics still left a signature in the ductal structure, such that the theoretical concentration of ducts close to the initial tip location was higher than at the more distant parts recently invaded.

Therefore, to define an alternative mechanism for tip termination, evidence from the experimental data was used to inspire a simpler mode of regulation. Based on the homogeneity of ductal density (Figure 1B), the scarcity of ductal crossovers and the proximity of inactive tips to existing ducts (Figure 1A), a revised model was considered in which active tips not only annihilate when they come in proximity of one another, but also terminate when they come into proximity of an inactive particle (i.e., the trailing ducts):

(6) $$A+I\to 2I,\;\text{at}\;\text{rate}\;r_{a}$$

Such a process complicated the classical problem of BARWs, making it fully non-Markovian: Understanding the dynamics of active walkers A required a knowledge of the full history of the random walk, and not just the current spatial configuration. Alternatively, one could hide the non-Markovian character of the problem in the second species I, which previously was simply slave to the active particles. This bore some resemblance to generalizations of directed percolation models in which a directed-percolation (DP) process is coupled to a frozen field. A key difference was there are then infinite numbers of absorbing states, as opposed to simply A=0. However, in the current model, the frozen particles I actively feedbacked on the dynamics of A, a situation reminiscent of two-species epidemic type models (Rossi et al., 2000). In the presence of process (6), the continuous mean-field approximation to the dynamics then modified Equation 5 to the form:

(7) $$\{\begin{array}{l} \partial_{t}a=D\nabla^{2}a+r_{b}a\left(1-\frac{a+i}{n_{0}}\right)\\ \partial_{t}i=r_{e}a+\frac{r_{b}}{n_{0}}a\left(a+i\right) \end{array}$$

where, for the sake of clarity, $a_{0}$ was renamed as $n_{0}$. One can rescale all times by $1/r_{e}$, all concentrations by $n_{0}$, and all distances by $\sqrt{D/r_{e}}$, and introduce the dimensionless ratio $\bar{r}=r_{b}/r_{e}$, so that the system of equations in one-dimension (coordinate x) became:

(8) $$\{\begin{array}{l} \partial_{t}a=\partial_{x}^{2}a+\bar{r}a\left(1-a-i\right)\\ \partial_{t}i=a+\bar{r}a\left(a+i\right) \end{array}$$

The parameter $\bar{r}$ could also be removed by introducing two different scales for the concentrations of a and i. However, this parameter was kept so that the magnitude of the two concentrations could be compared in a transparent manner in subsequent analyses.

To find a traveling wave solution for a and i, it was convenient to change coordinates to the co-moving frame by defining a variable z=x−Vt, where V is the wave velocity. The system of equations then became

(9) $$\{\begin{array}{l} -V\partial_{x}a=\partial_{x}^{2}a+\bar{r}a\left(1-a-i\right)\\ -V\partial_{x}i=a+\bar{r}a\left(a+i\right) \end{array}$$

Non-integrability of these equations prevented an analytical solution. Instead, it was useful to perform two approximations setting a≪i in the first equation and $\bar{r}\left(a+i\right)\ll 1$ in the second. This provided significant analytical insight into the dynamics, from which the two assumptions could be justified *a posteriori*. With these approximations, the coupled equations took the form:

(10) $$\{\begin{array}{l} -V\partial_{x}a=\partial_{x}^{2}a+\bar{r}a\left(1-i\right)\\ -V\partial_{x}i=a \end{array}$$

Compared to the classical Fisher-KPP equation, a difference was that the negative feedback did not arise from a, but from i, which was dynamically produced by a. This produced KPP pulses instead of KPP waves, with a having a single stable state a=0, and the pulse joining an a=0 front with an a=0 back (homoclinic orbit). However, the concentration of the “inhibitor,” i, adopted a different profile. The steady-state concentration for i was harder to read from Equation 10, but became obvious after substituting the second equation for a into the first, and integrating once, leading to:

(11) $$-Va=\partial_{x}a+\bar{r}i\left(1-\frac{i}{2}\right)$$

The constant of integration vanishes since all concentrations and their derivatives must vanish at x→∞. It then became more apparent that i=2, a=0 was a stable solution at the back at the wave, together with i=0, a=0 at the front. In contrast to the pulse shape of the profile of active tips a, the concentration of inactive ducts i thus adopted a traveling wave solution. From a physical perspective, this corresponds to the fact that active walkers can freely diffuse at the front of the wave, in the absence of inhibitors, but that they are trapped by inactive walkers at the back of the wave, and become inactivated. These processes specify a ductal network of well-defined, homogeneous, density i=2 at which the gland is patterned. Inserting the ansatz $a=Ae^{\lambda_{+}z}$ for the profile at the front of the wave into the linearized system of equations above, one obtained:

(12) $$\lambda_{+}=\frac{-V\pm \sqrt{V^{2}-4\bar{r}}}{2}$$

Similarly, at the back of the wave, with $a=Ae^{\lambda_{-}z}$, one obtained (noting that $\lambda_{-}$ must be strictly positive),

(13) $$\lambda_{-}=\frac{-V+\sqrt{V^{2}+4\bar{r}}}{2}$$

As $\lambda_{\pm }$ must be purely real, velocities below $V^{*}=2\sqrt{\bar{r}}$ cannot propagate. Moreover, a classical result of such KPP systems is that the velocity selected is exactly $V^{*}$, as verified through numerical integration of these equations (Figures S4D–S4G). Therefore, the decay length of the font and back of the pulse was given by

(14) $$\{\begin{array}{l} \lambda_{+}=-\sqrt{\bar{r}}\\ \lambda_{-}=\left(\sqrt{2}-1\right)\sqrt{\bar{r}} \end{array}$$

which predicted that the back of the pulse decays slower than the front. Concerning the validity of the approximations above, at the back of the pulse, i→2 and a→0, and the first approximation a≪i is trivially satisfied. At the front of the pulse, it followed from the expressions above that $a/i\approx 2\bar{r}$. However, $\bar{r}$ was the ratio of the timescale of tip elongation to tip branching. On a discrete lattice, this translated to the inverse of the average number of lattice steps which the walker explores before a branching event. Therefore, having a sparsely branched structure like the mammary gland imposed the condition $\bar{r}\ll 1$, as opposed to $\bar{r}\approx 1$, which would give rise to a dense structure, and the assumption a/i≪1 can be made with high precision. Similarly, $\bar{r}\ll 1$ allowed the validation *a posteriori* of the second approximation $\bar{r}\left(a+i\right)\ll 1$ (which, in fact, was exactly the same level of approximation as the first one, comparing $\bar{r}$ and O(1)). However, for the sake of completeness, numerical integrations of the full equations were also performed for $\bar{r}=0.1$ without approximation (Figures S4E and S4F), as well as numerical integrations for $\bar{r}=1$ (Figure S4G), when the approximation was invalid. These results verified that neither the phenomenology of the solution, nor the pulse asymmetry, are qualitatively affected.

#### Mammary gland parameters and numerical simulations

To confirm the validity of the analytical results obtained from the mean-field approximation to the model, full numerical simulations of the BARW were analyzed. From previous work on classical BARWs, mean-field theory was expected to be valid for small fluctuations (i.e., small annihilation probabilities), while the active state could be destroyed by fluctuations when they become large. A quasi one-dimensional front invasion was simulated by considering an elongated two-dimensional rectangular domain Ω of dimensions $L_{x}$ and $L_{z}<L_{x}$, so that $\Omega =\left\{\left(x,z\right)\in R^{2}:\left\{x\in \left[0,L_{x}\right],z\in \left[0,L_{z}\right]\right\}\right\}$. The initial condition was taken as a single active walker located at the left side of the domain $\left(0,L_{z}/2\right)$, with polarity facing the right/posterior side, p=(1,0). The length unit of the simulation was fixed by the elementary length of the random walk at each time point, l. Crucially, the most simple assumption for annihilation was taken so that an active particle (tip) terminates deterministically when entering into an annihilation radius $R_{a}$ of any active or inactive particle belonging to a different duct. Therefore, there was no need for implementing a definite rate of annihilation $r_{a}$ as it emerged from the spatial interactions. The timescale of the simulation was set as the time $\tau_{s}$ for each active particle to make an elementary step of length l=1 in the direction p. Therefore, the only truly free parameter of the numerical simulations was the branching rate $r_{b}$, which controls the final density of the ductal network, via the average branch length $l_{d}$. More precisely, the key parameter in this problem was the dimension of the fat pad, rescaled by the average branch length $l_{d}$. For the default set of parameters used to describe mammary gland morphogenesis, the experimentally measured rescaled long and short dimensions of the mammary gland (resp. $L_{x}/l_{d}$ and $L_{z}/l_{d}$, Figure S1D) were used. 2000 full stochastic simulations were ran, and the results averaged, providing parameter-free predictions, i.e., they did not involve the fitting of a free parameter.

Quantitative comparisons between the profiles of both a and i in the simulation agreed well with the analytical theory (Figures 3 and S4). In an unbound two-dimensional domain, as predicted by the KPP mean-field theory, numerical simulations displayed a characteristic pattern of radial expansion at a constant velocity (Figure S2A), with the active tips residing at the front of the invading wave. This produced a robust and characteristic convergence to a non-equilibrium steady-state network configuration (Figure S2B), on the timescale of a few generations, characterized by near-perfect balance between the rates of tip branching and termination.

The parameters for the mammary simulations were chosen as follows. Based on experimental measures (Figure 1), the rate of branching was set to $r_{b}=0.1$, together with box sizes of $L_{x}=280$ and $L_{y}=150$. At each time step (with step time τ=1 used as the unit time), tips were assumed to move forward by a length Vτ=1 (used as the unit length), along a polarity vector $p_{i}$ (specified in 2D by an angle θ), which diffused by a random angle of amplitude δθ=π/10. The annihilation radius was set to the characteristic width of a mammary duct, estimated at $R_{a}=3$ in simulation length units. Tips terminated if they passed the bounds of the simulation box. With these parameters, the model could predict with high precision the distribution of subtree size (defined as number of branches in a subtree, starting at branch level 6 from the embryonic rudimentary structure), as well as the subtree persistence (defined as the fraction of subtrees present at level 6 which are still active at a given level, or generation number). The broad nature of these distributions was a strong indication of the fact that active tips (and by extension different subtrees) compete neutrally: an ever-diminishing fraction of subtrees survives and colonizes an ever-expanding part of the fat pad.

#### Alternative models of mammary branching morphogenesis

To underline the singular predictive nature of the BARW model, one can consider the behavior of different models of branching morphogenesis, inspired by past proposals in the literature. The following methods sections shows that these alternative models are inconsistent with various aspects of the data, both qualitatively and quantitatively.

##### Fractal pattern

Fractal patterns have been previously proposed in the past to explain the branching morphogenesis of the lung (Iber and Menshykau, 2013) and human mammary lobule (Honeth et al., 2015), as a solution for the problem of space filling for exponentially increasing tip numbers. In the BARW framework, the problem is abolished by termination, which regulates the epithelial density. In the fractal branching concept, branch length and width diminish geometrically as a function of generation, so that an exponentially increasing number of branches can fit in a finite space. Importantly, there are key lines of evidence arguing against such a model in the mammary gland (as well as the kidney):

• The branching pattern is not observed to be deterministic (viz. subtrees do not show homogeneity in size) in these organs.
• The branching pattern cannot be explained by purely symmetrically dividing tips (Figures 2B–2D and Figure S1), i.e., the number of tips does not increase exponentially with generation number.
• Average branch lengths and widths are independent of generation number in the mammary gland (Scheele et al., 2017) (while they reach a plateau in the mouse kidney).

##### Branching and self-avoiding random walks

For kidney branching morphogenesis, a model of branching tips with self-avoiding properties was proposed and compared to the first branching events in *in vitro* experiments (Davies et al., 2014). This model avoids crossovers between ducts, thus reproducing a key aspect of the experimental data. However, a major contrast with the BARW framework is that the number of tips still increases exponentially with generation number, as there is no termination. Therefore, this model could not explain the data, as the branching statistics could not be explained by purely symmetrically dividing tips (Figure 2D). Moreover, one finds that a large degree of self-avoidance (see also Quantification and Statistical Analysis) increase significantly the anisotropy of the branching pattern, to values which exceed the experimentally measured mammary anisotropy (Figures S5F, S5G, and S5I). This points to the fact that tip annihilation must be a key feature of any viable model of branching morphogenesis. However, alternative sources of regulation/termination could be conceived, and are analyzed in detail below.

##### Regulation of branching

Branching, instead of termination, could be enforced in a density-dependent manner. From a mean-field perspective, these two options are formally identical and, therefore, cannot be distinguished. However, in full spatial simulations, the two hypotheses yield crucially different outcomes: Although a density-dependent regulation of branching could reproduce the constant average density of ducts (Figure S4A) as well as the pulse kinetics of invasion, it also yields numerous ductal crossovers (on the order of the number of branches, thus significantly overestimating the experimentally observed value of Figure 1C by an order of magnitude).

##### Stochastic termination, independent of spatial cue

Next, the frequencies of tip termination and branching could be encoded intrinsically, independent of any spatial information in the system. Termination was thus parametrized based on the experimentally measured data (Figure 2D), and implemented as a stochastic process (Scheele et al., 2017). This model failed to predict key qualitative aspects of the data. In particular, since it did not take into account spatial cues, it failed to reproduce the absence of ductal crossovers. Moreover, because of the absence of self-organizing properties arising from tip-duct interaction, active tips failed to self-organize into a sharp pulse of active tips at the edge of the tissue (see Figure S4B for a typical numerical integration), and were instead uniformly dispersed in the fat pad. Because invasion was not directional anymore, the trees failed to fill the entire fat pad, instead exploring the proximal part before stochastically terminating. Therefore, this led to a very poor fit to the angle probability distribution $\left(R^{2}=0.24\right)$. This strengthened the hypothesis that tip-duct interactions had to form the regulatory basis of any viable model of mammary branching morphogenesis. Models that keep tip-duct interaction in one form or another, to ensure that the ductal trees do not display crossovers, were thus subsequently explored, i.e., models that reproduced, at least qualitatively, some basic aspects of the data.

##### Branching and self-avoiding random walks with side-branching

A model based on a self-avoiding random walk with side-branching was first considered. This corresponded to introducing two “classes” of tips and branches: the main branches/tips were the same as above, and performed a branching and self-avoiding random walk, maintaining a sufficient spacing between them to avoid termination. However, a further assumption was of a constant probability $r_{s}$ to create side-branches at each branching event, i.e., tips that were unable to branch again, and terminated after a short typical length, regardless of the external environment. This was equivalent to termination being implemented “intrinsically” upon asymmetric branching, in opposition to the BARW framework above, where termination was considered as an extrinsic event based on local spatial rules and independent of the branching events themselves. Because of the self-avoiding nature of the dynamics, crossovers were avoided and, because of intrinsic termination, for $r_{s}=1$, the number of tips did not increase exponentially, and were thus intrinsically balanced. The possibility of mammary morphogenesis occurring sequentially was thus explored, in the spirit of proposals such as in (Huebner and Ewald, 2014): An early phase of largely symmetric branching served to increase the net number of tips, as observed experimentally, followed by a phase of purely asymmetric side-branching, with main branches avoiding each other. For robustness, two options were tested for the average threshold generation number to move to a phase of side-branching: generation n=5 (Figure S3G and blue curves in Figures S3I–S3K) and n=6 (Figure S3H and orange curves in Figures S3I–S3K), and averaged in each case over 1000 full stochastic simulations. The radius of repulsion $R_{a}=L_{x}/20$ and repulsion strength $f_{r}=0.6$ had to be taken large, as otherwise ductal crossovers occurred with high probability. With these parameters, one could obtain a satisfactory fit to the subtree persistence (Figure S3K). Importantly however, this model missed key aspects of the experimental data:

• At these levels of repulsion (which were necessary to avoid crossovers, as mentioned above), the simulated ductal trees were highly directional, with an angle distribution that fitted very poorly with the data (Figure S3I, $R^{2}=-4.1$ and $R^{2}=-2.8$ resp.), as it overestimated drastically the degree of directionality (see simulation snapshots).
• Moreover, because of the absence of strong competition between subtrees, the predicted subtree distribution fitted poorly with the experimental data (Figure S3J, $R^{2}=0.4$ and $R^{2}=0.7$, respectively), as it underestimated drastically the subtree size heterogeneity. Calculating $R_{log}^{2}$ provided an even worse correlation, as the tails of the model and data distribution diverged strongly.

##### Branching and annihilating random walks with external guidance

Next, a key alternative to the BARW model was considered, which involved the guidance of tips by external chemical gradients/cytokines, to explore the fundamental debate on whether morphogenesis proceeds via self-organizing features, or via decoding positional information. Specifically, the BARW framework defined above was supplemented by an additional external guidance, i.e., a biased instead of isotropic random walk. At each step of a tip i in a direction **p**_***i***_, an external field was imposed in the distal direction $g_{x}$ **x**, with $g_{x}>0$ (and the updated unit vector **p**_***i***_ in the presence of this field was calculated). Thus, increasing values of $g_{x}$ lead to more and more directional migration toward the distal direction. The effect of the perturbation became noticeable when $g_{x}$ was of a comparable order of magnitude to the rotational diffusivity of the tips (Figures S3A–S3C). Various values of $g_{x}=0.05,\;0.1,\;0.2$ were tested (respectively blue, orange and yellow lines in Figures S3D–S3F) and averaged in each case over 1000 full stochastic simulations. Importantly, this model also missed key aspects of the data:

• The main departure between simulation and experiment was, predictably, on the anisotropy of the branching pattern. For each value of $g_{x}$, as for the BARW simulations in the main text, the probability distribution of having a branch at an angle θ∈[0,π] from the horizontal **x** was computed. Crucially, this systematically overestimated the anisotropy, with increasing values of $g_{x}$ causing an increasing anisotropy, and providing a very poor fit for the data (Figure S3D, $R^{2}=-2.8$ for the best fitting case of $g_{x}=0.05$, thus a much worse fit than the control case of $g_{x}=0$).
• Moreover, the predicted subtree size distribution (Figure S3E) and persistence (Figure S3F) fitted increasingly poorly the experimental data (respectively $R^{2}=0.82$ to $R^{2}=0.56$ and $R^{2}=0.93$ to $R^{2}=0.83$, for $g_{x}=0.05$ and $g_{x}=0.2$). Calculating $R_{log}^{2}$ *i*ndicated an even worse fit, as the tails of model and data distribution diverged strongly.

##### Branching and annihilating random walks with side-branching

Next, the possibility of whether the data could accommodate a BARW framework coupled with significant side-branching was explored (defined as above as the generation with probability $r_{s}$ of tips that were unable to undergo further branching). Various values of $r_{s}$ were explored (all of the other parameters being the same as the default simulations from the main text) and in each case 1000 full stochastic simulations were averaged (Figures S3L–S3N). Importantly however, large values of $r_{s}$ again missed key aspects of the data. In particular, increasing values of $r_{s}$ caused a higher and higher value of the subtree persistence, as the perturbation decreased inter-tip competition. Therefore, the simulations with side-branching systematically overestimated subtree persistence (Figure S3O). At the same time, they also systematically underestimated subtree size heterogeneity (Figure S3P). Thus, including medium to large values of $r_{s}$ ($r_{s}=0.2$ in blue, $r_{s}=0.5$ in orange and $r_{s}=0.75$ in yellow) systematically worsened the fit of the model to the data ($R^{2}=0.91$ for persistence and $R^{2}=0.85$ for size distribution for $r_{s}=0.5$), allowing to rule out the possibility of a large fraction of side-branching during 3-8 weeks pubertal dynamics, although side-branching could play a more dominant role later on after the fat pad has been filled (Sternlicht, 2006).

##### Branching and annihilating random walks with large side-branching and repulsion

Finally, the question of whether a large amount of “secondary phase” side-branching could still accommodate the data with added repulsion was explored (again, in the presence of annihilation, as in the control simulation). The same two-phase model as above was implemented (symmetric branching followed by a phase of side-branching with probability $r_{s}$ after an average generation of n=6), with repulsion. A repulsion radius of $R_{a}=L_{x}/60$ and a repulsion strength $f_{r}=0.6$ were used (although these values were found to be less crucial as before, as crossover here was avoided in any case by the presence of annihilation). Again, to explore the phase diagram, various values of $r_{s}=0.75$, 0.9, 1 were tested (respectively blue, orange and yellow lines in Figure S3R) and 1000 full stochastic simulations were averaged in each case (see Figure S3Q for an example of a typical final configuration for $r_{s}=0.9$). Although this model reproduced rather well the orientation of the tree, as well as its constant spatial density and absence of crossovers, the key issue was again the low competition between subtrees, which caused an overestimate of subtree persistence, and an underestimate of the heterogeneity of the subtree size distribution (Figure S3R, $R^{2}=0.82$ for $r_{s}=0.75$ to $R^{2}=0.82$ for $r_{s}=0.9$ and $R^{2}=0.36$ for $r_{s}=1$). Once again, calculating $R_{log}^{2}$ indicated an even worse fit, as the tails of model and data distribution diverged strongly.

#### Theoretical analysis of bead implantation

There are three possibilities to theoretically simulate the effect of the beads with respect to the BARW framework: the beads could enhance locally termination (with the same termination radius for beads as ducts for the sake of simplicity); the beads could inhibit locally termination (so that, within the same radius, no termination could occur); or the beads could enhance branching (so that, within a critical radius, the branching rate was enhanced by a given factor $h_{b}$, with $h_{b}=2$ in Figure 5C, corresponding to the observed increase in branch rate, and $h_{b}=3$ in Figure S5L for comparison). In simulations, four clusters of 15 beads were randomly positioned, to mimic the experimental configuration. The probability of bead-duct crossover was computed in all cases, to compare to control. As shown in Figures S5L and S5O, in the control case of beads devoid of any effect, the probability for ductal crossover was slightly below 40%, whereas, by construction, the case of beads inducing termination there was a zero-probability for bead-duct crossover. Next, simulations were used to assess the influence of ectopic delivery of FGF10. Glands with FGF10 beads were compared with numerical simulations locally enhancing branching rate by a factor $h_{b}=2$ (Figure 5C), which reproduced well the enhanced branch density locally with minimal directionality.

#### Numerical simulations in 3D

To address the dynamics of the BARW in 3D, the model was parametrized in spherical coordinates (r,θ,ϕ), with the initial condition of a single active tip positioned at coordinate r=0 with orientation θ=π/2 and ϕ=0. Tips branch at a rate $r_{b}$ and terminate deterministically if they enter into a radius of $R_{a}$ of another duct. Upon bifurcation of a tip i, two offspring tips are produced, at an angle $\alpha_{i}$ from their ancestor duct. Based on previously published data on kidney branching, $\alpha_{i}$ was chosen randomly from a uniform interval $\left[\alpha_{o}-\delta \alpha ,\alpha_{o}+\delta \alpha \right]$, with $\alpha_{o}=50^{\circ }$ and $\delta \alpha /\alpha_{0}=1/3$ (Short et al., 2014). The results depended only very weakly on these parameters. Moreover, the two offspring tips adopted experimentally a diametrically opposite position, and the angle β∈[0,2π] for the plane of this bifurcation was thus chosen randomly (see Figure S6A for a schematic). The angles ($\theta_{1}$, $\phi_{1}$) of offspring 1 could thus be calculated from the angles (θ, ϕ) by solving the equations:

(15) $$\{\begin{array}{l} \text{cos}\theta_{1}\text{sin}\phi_{1}\text{cos}\theta \text{sin}\phi +\text{sin}\theta_{1}\text{sin}\phi_{1}\text{sin}\theta \text{sin}\phi +\text{cos}\phi_{1}\text{cos}\phi =\text{cos}\alpha_{i}\\ -\text{cos}\theta_{1}\text{sin}\phi_{1}\text{sin}\theta +\text{sin}\theta_{1}\text{sin}\phi_{1}\text{cos}\theta =\text{sin}\beta \text{sin}\alpha_{i} \end{array}$$

and the angles ($\theta_{2}$, $\phi_{2}$) of offspring 2 could be calculated by the same system of Equations (15) by substituting β→β+π. This guaranteed that there was both an angle of $\alpha_{i}$ between the directions of ancestor and offsprings, as well as that the two offsprings went in diametrically opposite directions relative to this angle.

Furthermore, as detailed in the main text, one must specify the anisotropy of kidney expansion in the simulations (Figures S6B and S6C), as this does not appear to arise from the redirection or annihilation of tips going along the short axis. Indeed, if that were the case, one would see, by analogy to the mammary gland expansion, that only tips along the long axis are proliferative. In contrast, both proliferation and nephrogenesis in kidney were found to be organized in continuous rims all across the surface of the developing organ (Costantini and Kopan, 2010). Anisotropy did not seem to be driven either by a much shorter branch length along the short axis, as the average branch lengths depended very little on the direction of growth (Figure S6E), whereas the branch length distribution was consistently well-fitted by a single exponential with a threshold (which was fitted from the experimental value, see theory versus experimental distributions on Figure S6F). Therefore, the simplest model of anisotropy was to assume that the timescale of the branching and elongation process depended on the angles (θ,ϕ) of a given branch relative to the center of mass, being rescaled by a factor F(θ,ϕ). $L_{z}$ denotes the longest axis (θ=0), $L_{x}$ the shortest axis (θ=π/2,ϕ=0), and $L_{y}$ the intermediate axis (θ=π/2,ϕ=π/2), which were measured experimentally as $L_{x}/L_{z}=0.5$ and $L_{y}/L_{z}=0.4$. Kidneys were thus parametrized as triaxial ellipsoids using the formula:

(16) $$F\left(\theta ,\phi \right)=L_{z}\sqrt{\frac{{\text{sin}}^{2}\theta {\text{sin}}^{2}\phi }{L_{x}^{2}}+\frac{{\text{sin}}^{2}\theta {\text{cos}}^{2}\phi }{L_{y}^{2}}+\frac{{\text{cos}}^{2}\theta }{L_{z}^{2}}}$$

Finally, although the global distribution of branch lengths in kidney was well-fitted by a single exponential (Figures S6D and S6F), indicative of a stochastic branching with constant rate $r_{b}$, it had been shown that the first bifurcations were more regular and stereotypic (Sampogna et al., 2015, Short et al., 2014), intervening dominantly in a single plane and along the future long axis of the kidney. To make accurate experimental predictions, a “seed” kidney was thus grown for n=5 generations of bifurcations, at a deterministic branch length of $1/r_{b}$, before using the stochastic branching dynamics described above. (Annihilation was still allowed in the early phase, with the same annihilation radius, for the sake of simplicity and consistency.)

#### Key parameters for the default kidney simulations

Numerical simulations were performed in three-dimensions. At each time step (with τ=1 again used as the unit time), tips moved forward by a length Vτ=1 (again used as the unit length), along a polarity vector $p_{i}$, or angle θ, which diffused by a random angle of amplitude δθ=π/10 (as in the case of mammary gland simulations). The branching rate was set to $r_{b}=0.2$ and a best-fit value for Figure 6D was found for an annihilation radius of $R_{a}=1.3$ in simulation length units (so that the only key parameter controlling the kidney morphology was $R_{a}^{\prime }=r_{b}R_{a}=0.26$). The bias toward ductal termination at the largest generation numbers was partially an artifact of the analysis, as these represented tips at the periphery of the kidney (Figure 6E) that remained proliferatively active but, without “progeny” yet, were counted as “terminated.”

#### Mapping between simulated and real embryonic time in kidney

To understand quantitatively the full developmental time course of embryonic kidney formation (as assayed experimentally by (Sampogna et al., 2015), the results of which were re-analyzed here), a correspondence between real and simulated time must be provided. The speed of kidney branching morphogenesis (defined as the characteristic time to form a branch) was found to decay in time (Sampogna et al., 2015) during embryogenesis (even though the average branch size decays slowly in time). Simulated time thus depended non-linearly on real embryonic time. For the sake of simplicity, constant time-invariant properties were considered in the simulations, and simulated time was then linked to embryonic time by fitting the average number of branches at that developmental time (from E11 to E19, see Figure S6G). All of the results presented in Figures 7A, 7C, and 7D were thus obtained using the simulated embryonic time correspondence shown on Figure S6G.

#### Modeling of Vitamin A deficient kidney

The analyses described above were applied to understand perturbations to normal kidney development. In particular, a dataset from the segmentation of the E15 mouse kidney on animals maintained on a low vitamin A diet (Sampogna et al., 2015) was re-analyzed. Interestingly, although the branching rate and total number of branches were maintained in this condition, kidney became much more heterogeneous with, in particular, an increase of the maximal branch generation number attained at E15 (some 3 generations more than wild-type, P<0.05), and thus a correspondingly decreased number of mid-generation branches. It was thus tested whether the same model could be applied to understand this new phenotype. Given the sensitivity analysis developed above, one needed to first explore whether a change of anisotropy in kidney shape could explain this behavior, but no significant changes was found in the kidney aspect ratio compared to wild-type (P>0.1 both for $L_{x}/L_{z}$ and $L_{y}/L_{z}$). However, a key geometric change was that Vitamin A deficient kidneys were much smaller, behaving as a spatially scaled down version of their wild-type counterparts (occupying on average 35% of normal wild-type volume, P<0.05). Therefore, as a consequence, the density of tips at the outer surface rim was found to be nearly twice as large in the Vitamin A deficient kidneys as wild-type. This meant that, although the branching rate was identical, the elongation rate of tips had to be reduced in Vitamin A deficient kidneys, leading to the hypothesis that this could explain, by itself, the enhanced heterogeneity, by increasing tip-tip competition. Indeed, for lower characteristic branch lengths, crowding-induced terminations were expected to arise earlier. It was thus checked whether this single geometric change in elongation rate of kidney could reproduce quantitatively the phenotypic differences between the wild-type and Vitamin deficient kidney. The elongation rate was thus decreased by a factor two in simulations, and keeping all other parameters constant, which lead to the correct reduction in kidney volume at E15 (Figures S7H and S7I for comparison of wild-type and mutant simulations). The same methods as before were used to computed the predicted average number of branches per generation, for the same total number of branches. Importantly, the simulations for Vitamin A deficient kidneys displayed on average two more generations than simulations for wild-type kidneys (Figure S7J), closely mirroring the data. Moreover, the model provided an overall excellent prediction ($R^{2}=0.98$, S=6) for the Vitamin deficient data. This validated the model of crowding-induced termination by showing that one can predict kidney structure from simple geometric properties such as average branch length. Interestingly, in the Vitamin A deficient kidneys, nephrogenesis was also impaired (Sampogna et al., 2015). This displayed an additional layer of complexity compared to the proposed framework, as the simplifying assumption was made in the main text to equate inactive tips with tips fusing to glomeruli to initiate nephron formation. This hinted, in particular, to the fact that crowding tip termination could be a distinct, not-fully overlapping property of nephron initiation, something that would need to be tested by combining large-scale reconstructions with EdU assays. Similarly, the above analyses neglected the subsequent re-organization, dilation and alignment of kidney ducts.

#### BARW from reaction diffusion equations

The framework of reaction-diffusion systems, which have been widely and successfully studied since Turing’s seminal work to understand collective biological phenomena, was used to provide clues on the emergence of BARW. It was assumed, following Turing, that a diffusible activator A both self-activated and activated another diffusible molecule I. This second molecule I was an inhibitor, negatively regulating A as well as itself. Under general conditions on the diffusion coefficients, this system was shown to generate arrested phase separation into complex motifs with a well-defined length scale (dots on a hexagonal lattice, stripes, reconnected labyrinths, etc.). This formalism was extended to show that the inclusion of a third molecular component, functioning as a substrate for the first two, allowed for the formation of branched structures (Meinhardt, 1982). Guo and colleague have revisited this paradigm (Guo et al., 2014) using the following four-species system, including an activator A, an inhibitor I, differentiated inactive cells Y, and a substrate S:

(17) $$\{\begin{array}{l} \partial_{t}A=c\frac{A^{2}S}{I}-\mu A+D_{A}\nabla^{2}A+\rho_{A}Y\\ \partial_{t}I=cA^{2}S-\nu I-D_{I}\nabla^{2}I+\rho_{I}Y\\ \partial_{t}S=c_{0}-\gamma S-\epsilon YS+D_{S}\nabla^{2}S\\ \partial_{t}Y=dA-eY+\frac{Y}{1+fY^{2}} \end{array}$$

where c, μ, $\rho_{A}$, ν, $\rho_{I}$, $c_{0}$, γ, ϵ, d, e and f are coefficients of interactions, and $D_{A}$, $D_{I}$ and $D_{S}$ are coefficients of diffusions (for details see (Guo et al., 2014)). Numerical analysis showed that this model is able to reproduce the different modes of branching morphogenesis observed *in vivo*. In particular, increasing the parameter ϵ, which quantified the consumption of the substrate by the epithelium, switched from a mode of side-branching to a mode of exclusive tip-splitting morphogenesis. Interestingly, performing numerical integrations of these equations for longer times showed that the branching pattern also displayed an annihilating property (Figure S7N).

### Quantification and Statistical Analysis

#### Quantifications

Whole-glands were manually reconstructed and using custom-made .NET software, the length and width of all the ducts, the coordinates of the branch points, the distance of the TEBs to the closest neighboring duct, and the fraction of EdU-labeling in the ducts and TEBs were scored. Statistical parameters including the exact value of *n*, precision measures (mean ± SEM or SD) are reported in the Figures or the Figure Legends. All other analyses and simulations were made using custom-made Python software. No randomization was used. For the quantifications of Figure 1 on fat pad/branch length and crossovers (i.e., ratio between ductal crossovers and the total number of branches in a gland), the analyses were performed on n = *14* glands (n = *7* fourth glands, n = *7* fifth glands) of comparable size from 7 different 8 week old mice (full dataset). For the quantifications of Figure 2 on subtree size and persistence, the analyses were performed on n = *12* glands of comparable size from 6 different 8 week old mice (2 glands from one mice were excluded from this analysis, in order not to artificially enhance subtree heterogeneity, since their fat pad size was more than two SD away from the average size). For the quantifications of Figure 3D, the analyses were performed on n = *4* glands (n = *2* third glands and n = *2* fourth glands) of comparable size from two different 5 week old mice after a 4 hour Edu pulse. For the quantifications of Figures 4A and 4B on density fluctuations, the analyses were performed on n = *14* glands from 7 different 8 week old mice (full dataset). For the quantifications of Figures 4C and 4D on branch anisotropy, the analyses were performed in all glands in which an antero-posterior axis could be defined (n = *7* glands from 5 different 8 week old mice, with n = *5* fourth glands, n = *2* fifth glands), and branches inside a rectangular box around this axis were quantified (dashed gray line on Figure 4C), which allowed us to avoid the data becoming corrupted by the shape of the gland. For the quantifications of Figures S5O and S5P, the analyses were performed on n = *4* mammary glands for TGF-beta beads from 2 different mice, n = *3* glands from 2 different mice for the control beads, and n = 3 glands from 2 different mice for the FGF10 beads. For the quantifications of Figures 7A and 7B, the analyses were performed in n = *3* kidneys from 3 different embryos at each time point. For the quantifications of Figures 7C and 7D, the analyses were performed in n = *3* kidneys from 3 different embryos at each time point, except at E18-19 (n = *2* kidneys from 2 different embryos). It should be noted that for the latest time point of kidney development, the data allowed for the counting of nephrons and branches (Figures 7A and 7B), but could not always be used to reliably reconstruct connectivity, so that E16-17 and E18-E19 kidneys were grouped for enhanced statistics (resp. n = *3* and n = *2* kidneys from 3 and 2 different embryos). For Figure 7E, density fluctuations in n = *3* kidneys from 3 different E17-E19 embryos were averaged. For Figure 7J, n = *4* kidneys from 2 different E15 embryos were averaged.

#### Goodness-of-fit statistics

To challenge quantitatively the goodness of the fits in the main text, as well as its explanatory power, the model predictions were challenged by calculating in both cases the coefficient of determination $R^{2}$, the simplest indicator of the goodness of a fit. In addition, the standard error of the fit, S, was calculated, an absolute measure of the residuals of the fit, which has been shown to be more adapted for non-linear fitting procedures. For a good fit, $R^{2}$ should be as close to 1 as possible, whereas S should be as close to 0 as possible. Values of $R^{2}$ were also calculated for completeness by taking first the logarithm of the data and predictions (which was denoted as $R_{log}^{2}$ in the main text), given the power laws seen in several quantities of the dataset. This measure was complementary, as it gave comparatively more weight to the agreement between theory and experiments for small parameter values. Importantly, it was checked that one obtained satisfactory goodness of fits measurements in each case. Specifically, for the definition used in the main text, defining n as the number of the points being fitted, $\bar{y}=\left(1/n\right)\sum_{i}^{n}y_{obs}^{i}$ as the average of the observable, $S_{tot}=\sum_{i}^{n}{\left(y_{obs}^{i}-\bar{y}\right)}^{2}$, and $S_{res}=\sum_{i}^{n}{\left(y_{obs}^{i}-y_{model}^{i}\right)}^{2}$, the coefficient of determination was defined as $R^{2}=1-\left(S_{res}/S_{tot}\right)$, while the standard error of the fit was given by $S=\sqrt{\left(S_{res}/n\right)}$. As described below, for the shaded areas of Figures 2E and 2F, numerical integrations were performed for the experimentally observed average value of the branch lengths, as well as for the values ± one standard deviation. For all three values of resulting $r_{b}$ estimate, at least 2000 numerical simulations were performed and curves were computed for each case. The shaded area thus represented the sensitivity of the model prediction with respect to one standard deviation variation of the key parameter $r_{b}$. Moreover, to build the error bars shown in Figures 2E, 2F, 7C, and 7D for the experimental values of the cumulative subtree size distribution and subtree persistence, given the small number of subtrees in each mammary gland, a bootstrapping method was used. This involved calculating the cumulative distribution function for a large number of artificial datasets, which were samples with replacement of the original dataset (i.e., sizes or maximal generation, respectively, of subtrees), and calculating error bars, defined here as confidence intervals of one standard deviation, from the resulting cumulative distributions of each artificial dataset. For the shaded areas of Figures 7A and 7B, at least 1000 numerical simulations were performed using the default parameters (see above) and the branch number per generation and inactive versus total tip number were calculated in each case. Averages and 95 % confidence intervals were computed from these simulations, which therefore expressed the stochastic fluctuations of the model for a given and constant value of the annihilation radius $R_{a}$.

#### Statistics for bead experiments

To quantify the effect of beads on the branching pattern, as well as its locality, the average branchpoint-to-branchpoint distance was measured in four settings: in regions close and far from the beads (defined as regions which are closer/further than 500μ m to a bead), both for control beads and soaked beads. Importantly, when comparing glands with control beads versus glands with FGF10 beads (n=3 glands for FGF10 beads, n=3 glands for control beads), no statistically significant difference was found between the branch length far from control beads, in proximity to control beads, or far from FGF10 beads (adjusted P>0.99 in all cases, from a Kruskal-Wallis test with multiple comparisons and correction). However, the branches close to FGF10 beads were statistically much shorter (approximately twice as short, P<0.001 from a Kruskal-Wallis test, in all comparisons with the three controls).

#### Sensitivity analysis on mammary gland simulations

The overall robustness of your model with respect with parameter variations was explored, showing, in particular, that its predictions were insensitive to details of the simulation scheme used.

##### Effect of the branching rate

First, the effect of variations in the branch length, i.e., changes in the branching rate $r_{b}$, were shown in the main text as confidence intervals. The experimentally measured standard deviation of the mean branch length among different mammary glands was used, and thus branching morphogenesis for $r_{b}=0.085$ and $r_{b}=0.12$ was simulated, to build confidence intervals of one standard deviation with respect to the branching rate (shaded area in Figures 2D–2F). Lowering (respectively increasing) the branching rate increased (respectively decreased) the average number of branches in a mammary gland, and thus shifted significantly both the subtree size distribution and persistence (Figures 2E and 2F, shaded area). Interestingly, it was found to have relatively little impact on the convergence toward balance between termination and branching (Figure 2D). Moreover, lowering (respectively increasing) the branching rate modified the exponent of the giant number fluctuations (Figure S5C and main text): Larger values of the branching rate tiled space more effectively, so that the exponent was reduced, indicative of lower fluctuations. Conversely, smaller values of the branching rate approached the critical point, enhancing density fluctuations.

##### Effect of the annihilation radius

Second, the sensitivity of the results with respect to the value of the annihilation radius was investigated. This parameter was not expected to be crucial in two-dimensions for persistent random walks, as the probability for two lines to intersect is unity. Nevertheless, to confirm this intuition quantitatively, numerical simulations were run for different values of the annihilation radius $R_{a}$ (smaller value of $R_{a}=1.5$ shown in Figure S2D and larger value of $R_{a}=3.75$ shown in Figure S2E). Lowering the annihilation radius slightly delayed the convergence toward balance between tip branching and termination (Figure S2F), but this effect was found to be very small, even for more than two-fold variations in the annihilation radius $R_{a}$. Similarly, the cumulative subtree size probability was largely unaffected by large differences in $R_{a}$ (Figure S2G).

##### Effect of the persistence length

Third, the sensitivity of the results was investigated with respect to the value of the persistence length for the random walk. As stated above, a control value of δθ=π/10 was used, based on previous measurements of duct “tortuosity,” i.e., the average real path length between consecutive duct branch points, $L_{d}$, was 6% larger than its straight branchpoint-to-branchpoint value $l_{d}$ (Paine et al., 2016). For small path fluctuations, h(x), with respect to the branchpoint-to-branchpoint axis x, $\left(L_{d}-l_{d}\right)/l_{d}\approx h^{\prime }{\left(x\right)}^{2}/2\approx \delta \theta^{2}/2$; hence the value chosen. Again, numerical simulations were run for different values of the persistence length, by varying the magnitude of the angle variation at each step of the random walk, δθ. Both the limit of an infinity persistent walk (δθ=0, Figure S2H), and a slowly persistent random walk, with an angle δθ doubled compared to its reference value ($\delta \theta =2\delta \theta_{ref}$, Figure S2I), were considered. Although the microscopic morphology of the simulated glands was found, as expected, slightly different, these parameter changes did not significantly affect either the evolution of the tip termination probability as a function of generation (Figure S2J), nor the cumulative subtree size probability (Figure S2K). This argued for the robustness of results with respect to even large perturbations of this parameter.

##### Effect of probabilistic annihilation

Fourth, although the mammary gland reconstructions indicate that the crossover between ducts was a rare event, it was not non-existent, as assumed so far in the simulations. The simulations were thus modified to allow for the possibility of crossovers, and implemented probabilistic annihilation:

(18) $$A+I\to 2I,\;\text{at}\;\text{rate}\;r_{a}$$

if an active tip A entered the vicinity of a duct, i.e., within the annihilation radius, $R_{a}$. The reference simulation was thus simply the limit of $r_{a}=1$. In Figure S2L, $r_{a}=0.2$ and $R_{a}=5$ were chosen (i.e., a larger annihilation radius than the reference case, to compensate for the less likely annihilation, and to obtain simulated glands of comparable density). Although simulations now presented a low but non-zero fractions of crossovers (Figure S2L), the convergence toward a balance between termination and branching was still observed (Figure S2N), with similar kinetics. Interestingly, the functional shape of the cumulative subtree size distribution was characterized by decreased heterogeneity, with less very large subtrees compared to the reference distribution (Figure S2O). This resembled the distribution previously found using a mean-field limit of this problem, i.e., exponential tails, arising from the stochastic choices of equipotent tips in a critical Galton-Watson birth-death type process (Scheele et al., 2017). This was not surprising, as in the limit of very large annihilation radii, the spatial advantage for a tip to be in a crowded region becomes vanishingly small, converging toward the mean-field solution.

##### Effect of the branching angle distribution

Fifth, the importance of the branching angle distribution in dictating the final morphology of the mammary gland was investigated. In the reference simulations, the input for the branching angle distribution was its experimentally observed counterpart. Indeed, it was found that the branching angles of the two offsprings relative to the direction of the ancestor branch did not show any significant correlation, although the relative angle between the two off-springs were above a minimum bound of roughly π/16. In the simulations, one can thus stochastically and independently choose both from a Poisson distribution of parameter π/6 (together with a minimal bound of π/16). However, to also assess whether these choices of the branch angle distribution modified significantly results, simulations were modified by considering the branching angle between ancestor and offspring to be a given constant of α=50 degrees. This yielded a slightly more ordered gland morphology (Figure S2M). Importantly, however, the convergence toward a balance between tip termination and branching (Figure S2N), as well as the cumulative distribution of subtree sizes, were only very weakly affected (Figure S2O).

##### Effect of additional self-avoidance

Finally, it was assessed whether and how the results would be modified if tips had additional self-avoiding properties (Sternlicht, 2006, Davies et al., 2014), in addition to their previously-described branching and annihilating properties. Self-avoidance was modeled microscopically in the simplest local way, by assuming that a particle, located at a position **r**_***i***_, could sense an average density vector $p_{i}^{r}$ arising from ducts and tips in a repulsion radius $R_{r}$ (sketched on Figure S5E) such that,

(19) $$p_{i}^{r}=\frac{\sum_{\begin{array}{c} j\\ \left|r_{i}-r_{j}\right|<R_{r} \end{array}}\left(r_{i}-r_{j}\right)}{\left|\sum_{\begin{array}{c} j\\ \left|r_{i}-r_{j}\right|<R_{r} \end{array}}\left(r_{i}-r_{j}\right)\right|}$$

$p_{i}^{r}$ thus being a unit vector weighting equally every particle in a radius of $R_{r}$. At each random step of the random walk with l=1 in the direction $p_{i}$ (as described above), the particle performed an additional displacement of $-f_{r}p_{i}^{r}$ (with its polarity vector $p_{i}$ being updated accordingly). Thus, positive values of $f_{r}$ corresponded to self-avoiding random walks (i.e., tips moving away from denser regions), while negative values of $f_{r}$ corresponded to self-attracting random walks. $f_{r}$ represented the strength of the self-avoidance bias on the random walk. Moderate ($f_{r}=0.2$, Figures S5E–S5J) biases were investigated to understand whether this affected the results. Boundaries could also be represented formally as ducts (with the same particle density), and included in Equation 19, if one wanted to assume that the boundaries of the mammary fat pad repel active tips ($f_{r}=0.2$, Figure S5G). One must have $R_{r}>R_{a}$ in the simulations, i.e., a larger self-avoidance radius than the self-annihilation radius, otherwise tips terminated before they got a chance to sense their neighbors, and adapted their trajectories accordingly. In the simulations of Figures S5F–S5J, $R_{r}=2R_{a}$ was used. Simulated glands with higher degrees of repulsion were found to grow to larger subtree sizes and density, as expected from their repulsive properties, which allowed them to explore space more efficiently before terminating. However, the kinetics of convergence toward balance of tip termination and branching were only very slightly delayed (one generation, Figure S5H). This was found to be in large part because, in two-dimensions, particles still annihilated at comparable rates given the high probability of crossings. However, one quantitative change arising from repulsion was the enhanced emergent anisotropy of the branching random walk. To quantify this, the same quantifications of branch angle relative to the horizontal were performed, as in the reference simulation (Figure 4C), and the reference angle distribution was compared with the distributions in the different repulsive conditions. For small $f_{r}$, it was found that the bias toward distal orientations (angles close to 0) increased with increasing repulsion $f_{r}$. Moreover, including the repulsive effect of the boundary increased the bias even further (Figure 4D). This was expected, as boundaries now “guided” the tips in the proximal-distal direction via repulsion. Interestingly, this thus predicted anisotropies which were larger than the average experimentally observed value, arguing that this should not be a key feature of the data. Moreover, the reference simulation already achieved a relatively large value of anisotropy in the absence of any repulsion. This indicated that self-avoidance reinforced the emergent effects that was observed from branching and annihilating random walks, but were not necessary qualitatively and quantitatively to explain them. Of note, the dataset did present some inter-gland heterogeneity in the branch angle distribution anisotropy, with some glands displaying much more directional invasion than others. This could potentially be explained by various strengths of the self-avoidance properties in different glands. Finally, self-repulsion was found to decrease density fluctuations (Figures S5F and S5G), which manifested quantitatively by a reduced exponent of giant number fluctuations (Figure S5J), as expected from an ordering repulsive mechanism.

#### Kinetics of mammary gland invasion

As shown on Figure 3, although the bulk of proliferative cells were found to be localized in the invading front, one could also observe a fraction of tips which still contained a few proliferative cells, and were localized further away from the invasion front. However, these tips were always found small and less proliferative than at the front, lacking the characteristic “balloon shape” of front tips (Figure S4I). These small “half-active” tips were typically localized at the edge of the fat pad. One could thus conjecture that they represent formerly active tips during their transition to termination, and they were added to the description (which was formally equivalent to describing the localization of newly formed nephrons in Kidney, as shown in Figure 6D and discussed below) by writing an additional equation for their concentration h as:

(20) $$\{\begin{array}{l} \partial_{t}a=\partial_{x}^{2}a+\bar{r}a\left(1-a-i-h\right)\\ \partial_{t}h=a+\bar{r}a\left(a+i+h\right)-r_{h}h\\ \partial_{t}i=r_{h}h \end{array}$$

where $r_{h}$ described the rate of transition between half-active and inactive particles. Within this framework, h adopted a spatial shape similar to the one of active tips (as its equation was slave to a as soon as h≪i, which was typically the case). Although $r_{h}$ could be fit from the spatial distribution of half-active tips, its inclusion over-complicated the analysis so that, in Figure 3D, only fully active tips were considered, i.e., tips which consisted of over 50% of proliferative cells (as assessed by EdU measurement). Importantly, even with this conservative definition of active tips, one still recapitulated a key prediction of the model regarding the asymmetry of the pulse: the back decay was much slower than the front decay. Moreover, although the model slightly underestimated the density of ducts and active tips (Figure 3D), it was found to still provide a very good prediction for the detailed shape of both, with exponential decays on both sides of the pulse. For the simulations underlying the theoretical curves of Figure 3D, the relative sizes of the fat pad were fitted by measuring it once again for the n=4 glands used in the EdU experiment, as these were larger than the previous dataset (by around 60%), all other parameters being kept constant. Again, however, this did not change the conclusion nor the simulated profile shapes of the glands.

#### Giant density fluctuations in kidney

In order to assess the scale of giant number fluctuations in the experimental data, without the measure becoming corrupted by boundary effects, only detailed reconstructions of E17 to E19 kidneys were examined (n=3, average shown on Figure 7E; results for different time points were consistent, as indicated by the small error bars). The statistical analysis was then performed in a rectangular three-dimensional box around the center of mass of each kidney, thereby excluding boundary effects. Results consistently showed robust power law dependences with exponents larger than 0.5, indicative of giant number fluctuations. The same analysis was performed for the density fluctuations of the simulated kidney, on E19-equivalent trees, again avoiding the edges of growth to prevent boundary effects from corrupting the data.

#### Sensitivity analysis on kidney simulations

A sensitivity analysis of 3D BARW was performed, to understand how variations in the parameters of the model affected quantitatively and qualitatively the observed results for kidney morphogenesis.

##### Effect of the annihilation radius

In contrast to the case of two-dimensional BARWs, the value of the annihilation radius $R_{a}$ was found to play a crucial role in three dimensions. This was because vanishingly small radii $R_{a}$ gave rise to vanishingly few crossovers/terminations in three dimensions. Thus, the radius $R_{a}$ became a key parameter, and had to be fitted with respect to the kidney data in order to be able to make quantitative predictions. First, all annihilation events were suppressed $\left(R_{a}=0\right)$ to check whether the heterogeneity of the branch level distribution could stem from purely stochastic branching and size anisotropy. Importantly, this provided a very poor fit to the data (first panel, Figure S6J), showing the importance of annihilation for heterogeneity. Next, the same three-dimensional simulation as in the control were performed for large $\left(R_{a}=0.5R_{a}^{ref}\right)$ or small $\left(R_{a}=2R_{a}^{ref}\right)$ values of the annihilation radius (respectively center left and center right on Figure S6I, to be compared to the left panel for control). Larger radii enhanced the heterogeneity of the simulated kidneys, as was seen by a broadening of the segment distributions as a function of generation and at different developmental timings (second panel on Figure S6J). This translated into larger nephron to active tip ratio (Figure S6K), largely overestimating the experimentally observed values. Conversely, smaller radii were found to decrease the heterogeneity of the simulated kidneys (third panel on Figure S6J), and underestimate the nephron to active tip ratio (Figure S6K). This confirmed the importance of this parameter, and argued that it could be estimated rather precisely.

##### Effect of additional self-avoidance

Although the model performed well to reproduce key features of the kidney structure, topology and nephrogenesis pattern, as is obvious qualitatively from the three-dimensional reconstructions, and quantitatively from the giant density computation of Figure 7E, it overestimated the spatial density fluctuations, so that experimental reconstructions were consistently more ordered than their simulated counterpart. As mentioned above, it had been proposed that kidney has self-avoiding properties (Davies et al., 2014), in a Bmp7-dependent manner, as inhibition of Bmp7 function in cultured kidneys causes collisions between tips and ducts. However, another interpretation of this data, could be that collisions are avoided instead by termination, rather than repulsion. Notably, evidence in favor of this was obtained from culture studies of two kidney buds in close proximity (reproduced in Figure S6M from (Davies et al., 2014)). Indeed, when two trees growing in a similar geometry to the experiments were simulated, both for the case of pure termination without repulsion (Figure S6N) and pure repulsion without termination (Figure S6O), the simulations with termination were found to reproduce better the presence of numerous (terminated) tips in the contact zone between the two kidneys, whereas the pure repulsion simulations displayed an absence of tips at the contact zone, as they avoided the zone by adoption of diverging flow motion. However, to expand our analysis, it was asked whether, when applied to the *in vivo* kidney data, a degree of self-avoidance coupled to the BARW framework would improve the theoretical predictions. As the avoidance strength $f_{r}$ was increased, the number of annihilation events was found to diminish, and the branching topology thus became more deterministic, and characterized by more peaked distributions. However, this could be counteracted by increasing in concert the annihilation radius $R_{a}$. In particular, it was found that for an annihilation radius of $R_{a}=2R_{a}^{ref}$ and $f_{r}=0.3$ together with a repulsion radius of $R_{r}=6$, all other parameters being maintained the same (see Figure S6I for a typical simulation output showing higher order than control simulations), good fits for the nephron versus tip number as a function of time could still be obtained (Figure S6L), as well as for the number of branches per generation distributions at all time points (Figure S6J, bottom). Moreover, in these simulations, the spatial variations in density were markedly reduced (Figure 6E), so that the exponent of the giant number fluctuations observed experimentally could be predicted with much better accuracy compared to the non-repulsive case.

##### Effect of the growth anisotropy

Next, the contribution of the anisotropy in promoting subtree heterogeneity in the kidney was assessed. Numerical simulations were thus performed with the same set of parameters as wild-type, only in isotropic growth conditions ($L_{x}=L_{y}=L_{z}$, Figure S7A). As expected, the key phenomenology of a self-organized pulse of active tips at the edges of the kidney was not affected by changes in isotropic conditions (Figure S7A). Moreover, similar kinetics and scaling laws in the number of nephrons versus tips as a function of time were observed. However, a key difference was observed in the number of branches per generation as a function of time, with a marked reduction in the width of the distribution, as expected from reducing the anisotropy-induced growth advantage of subtrees in favorable directions (Figure S7B).

##### Proximity to the critical point

Finally, how the stochasticity of branching morphogenesis could lead to a stochastic transition to a fully annihilated state was assessed systematically. Indeed, within the mean-field theory, annihilation of the entire tree could not occur, as any non-vanishing value of the branching rate were sufficient to yield a steady state non-zero density of active tips. However, fluctuations, when taken into account through the full numerical simulations, were found to be able to destroy this active steady state, thus giving rise to a non-zero probability for full tree annihilation. The frequency of termination was thus varied by performing a parameter sweep in the annihilation radius $R_{a}$. (Although the branching probability $r_{b}$ could also have been used as a converse variable, this was computationally more intensive as it required simulating many particles for the low branching rate limit.) The same parameter set as in the control kidney simulations was used (i.e., non-repulsive), although three-dimensional isotropic simulations without a deterministic seed were performed here in order not to confound the analysis. For obvious technical reasons, trees cannot be simulated for an infinite amount of time, so that finite-size effects were expected, i.e., full annihilations which might occur later than the threshold simulation time would be discarded. However, the frequency of such events became vanishingly small in time, and only mattered in the very close vicinity to the critical point. The criteria chosen was that trees reaching 100,000 particles were considered non-annihilated/survivors, at least 1000 simulations per parameter value were run (see Figures S7E–S7G for representative examples), and tree survival probability was calculated as a function of the annihilation radius. Importantly, and in qualitative agreement with the literature (Cardy and Täuber, 1996), it was found that above a threshold in the annihilation radius $R_{a}$, the tree survival probability vanished, while it continuously increased below this threshold, indicative of a continuous phase transition (Figure 7F). Below a secondary lower threshold, survival always occurred. Interestingly, as could be expected from a biological perspective to avoid tree extinction, both these thresholds were found to be larger than the control value used to fit the *in vivo* kidney data (the same was true of mammary glands and two-dimensional simulations), by ratios of 2 and 3, respectively (Figure 7F, dashed vertical line).

## Author Contributions

E.H., C.L.G.J.S., J.v.R., and B.D.S. conceived the study and designed the experiments. C.L.G.J.S., M.M., R.H., N.D., and R.V.S. performed experiments and contributed data. E.H., C.L.G.J.S., M.M., and R.V.S. performed analyses. E.H. and B.D.S. developed the theoretical framework. C.L.G.J.S. and E.H. made the figures. J.v.R. and B.D.S. supervised the study. All authors discussed results and participated in the preparation of the manuscript.

## Supplemental Information

Movie S1. Typical Simulation Output from the 2D Numerical Simulations Using the Mammary Control Parameters, Related to Figures 2 and S2 Ducts are shown in black, active tips in red.

Movie S2. Typical Simulation Output from the 3D Numerical Simulations Using the Kidney Control Parameters, Related to Figures 6, 7, and S6 Ducts are shown in black, active tips in red and inactive tips in yellow.

Movie S3. Typical Simulation Output from the Isotropic 3D Numerical Simulations Used to Build the Phase Diagram of Figure 7F, Related to Figures 7 and S7 Here, we have used *R*_*a*_=3.8, i.e. just below the critical point. Ducts are shown in black, active tips in red and inactive tips in yellow.
