# Supplementary figures and images for: A Unifying Theory of Branching Morphogenesis
Source: Cell. 2017 Sep 21;171(1):242–255.e27. doi: 10.1016/j.cell.2017.08.026 (PMC5610190; doi:10.1016/j.cell.2017.08.026)

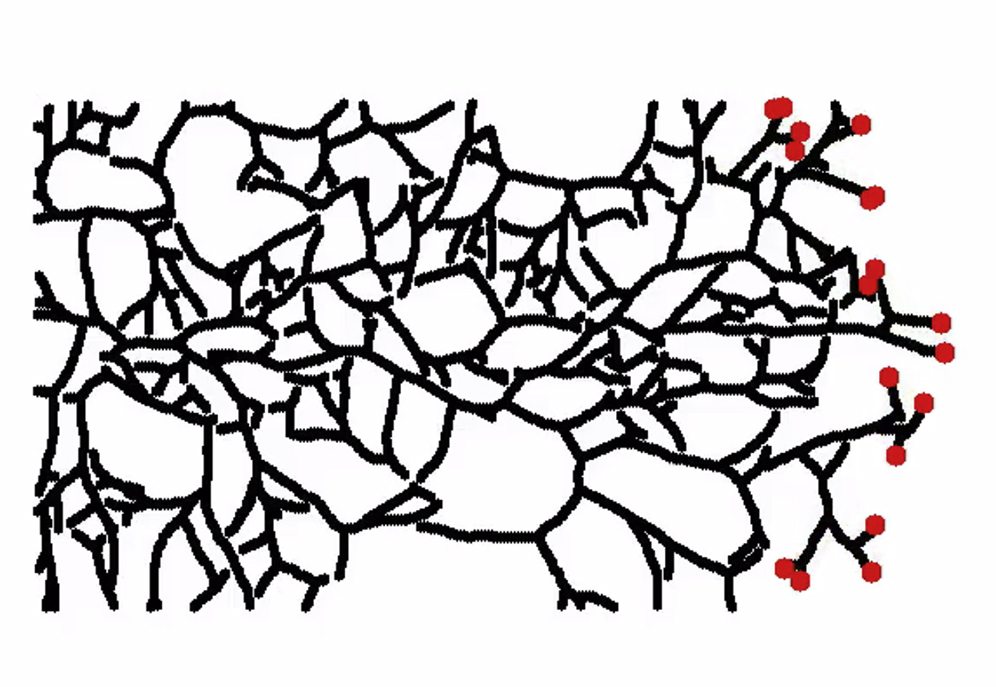

Supplement: Movie S1. Typical Simulation Output from the 2D Numerical Simulations Using the Mammary Control Parameters, Related to Figures 2 and S2 — Ducts are shown in black, active tips in red. [file mmc1.jpg]

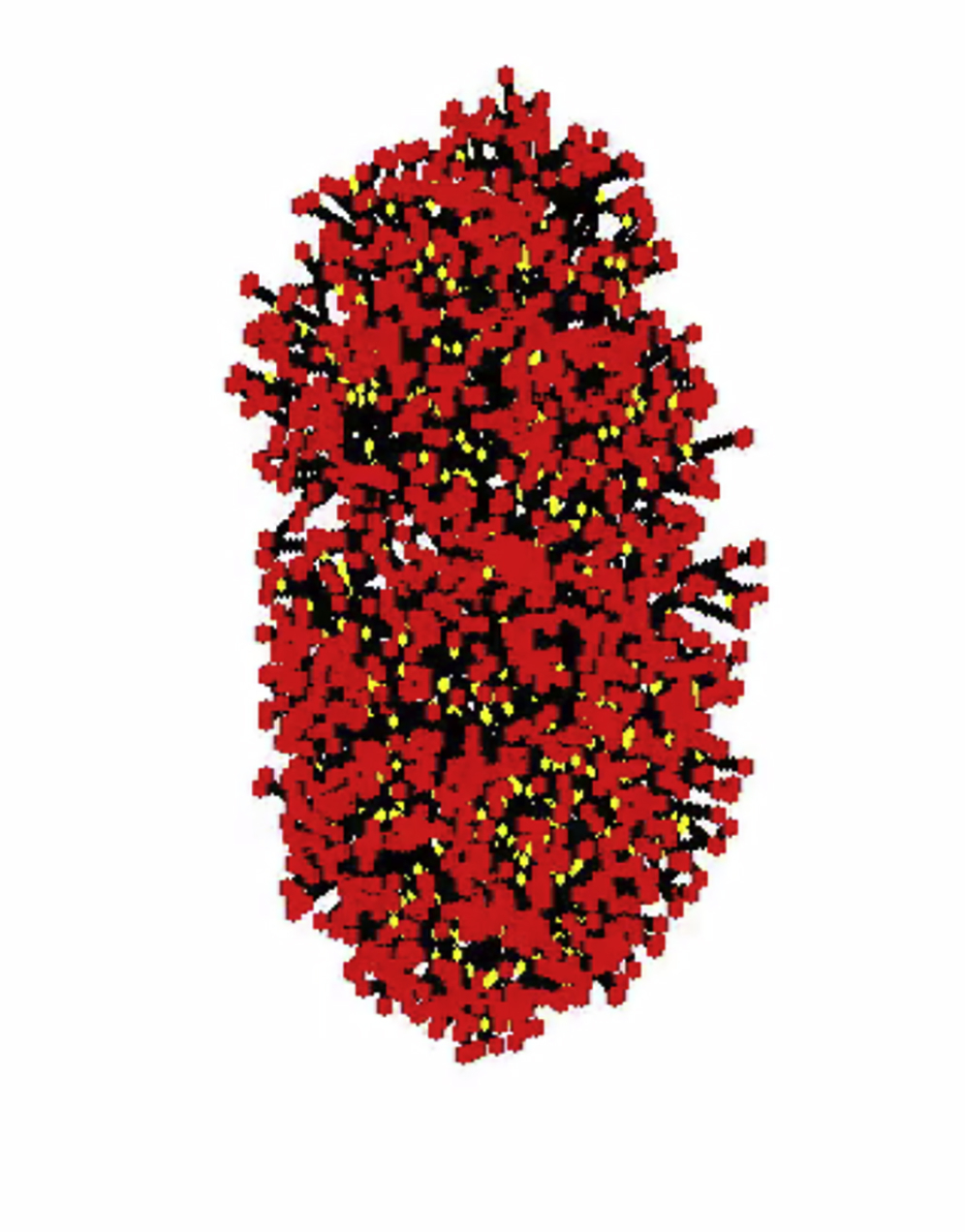

Supplement: Movie S2. Typical Simulation Output from the 3D Numerical Simulations Using the Kidney Control Parameters, Related to Figures 6, 7, and S6 — Ducts are shown in black, active tips in red and inactive tips in yellow. [file mmc2.jpg]

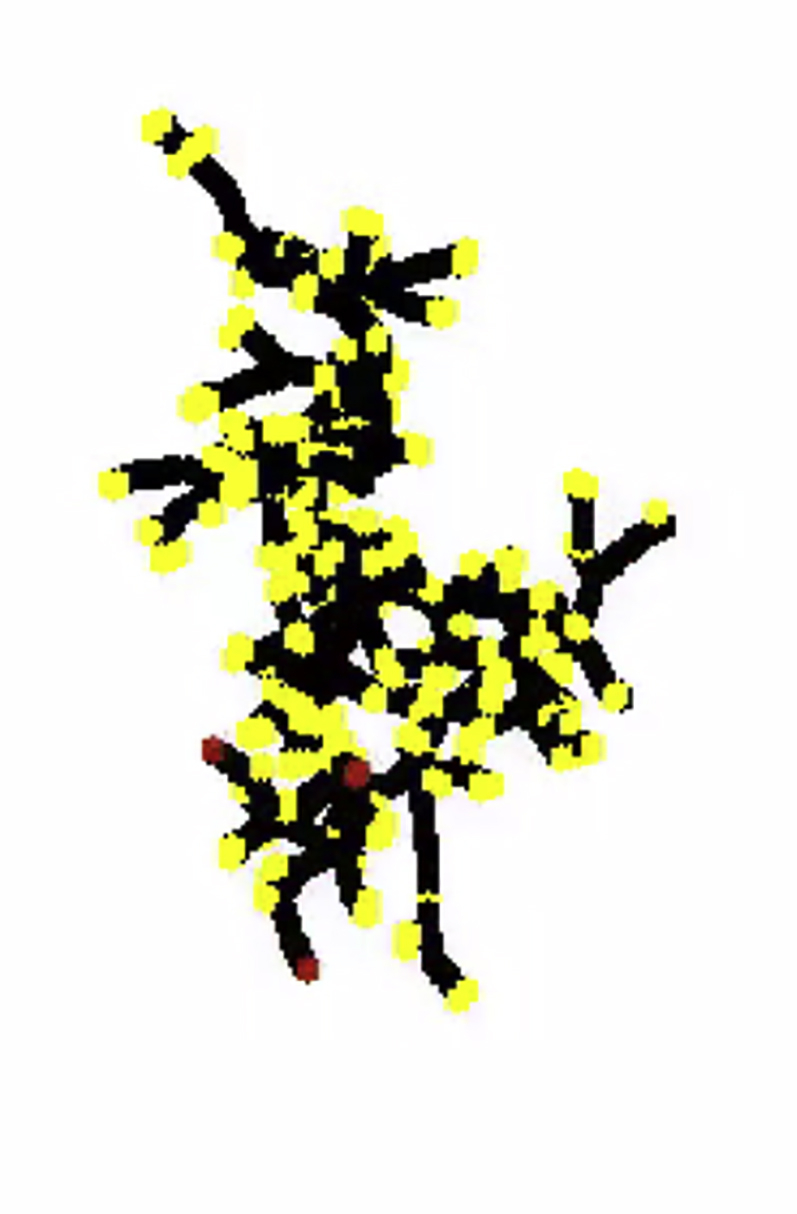

Supplement: Movie S3. Typical Simulation Output from the Isotropic 3D Numerical Simulations Used to Build the Phase Diagram of Figure 7F, Related to Figures 7 and S7 — Here, we have used Ra=3.8, i.e. just below the critical point. Ducts are shown in black, active tips in red and inactive tips in yellow. [file mmc3.jpg]
